# Synthesis and Biological Evaluation of Novel Piperidine-3-Carboxamide Derivatives as Anti-Osteoporosis Agents Targeting Cathepsin K

**DOI:** 10.3390/molecules29174011

**Published:** 2024-08-24

**Authors:** Yali Wang, Ting Guan, Hegen Xiong, Wenxin Hu, Xianjian Zhu, Yuanyuan Ma, Zhiqing Zhang

**Affiliations:** 1 School of Pharmacy and Life Sciences, Jiujiang University, Jiujiang 332005, China; 19196726989@163.com (T.G.); 13576831960@163.com (H.X.); 13979266920@163.com (W.H.); 18720459169@139.com (X.Z.); 15079428598@163.com (Z.Z.); 2 Institute of Medicinal Biotechnology, Chinese Academy of Medical Science and Peking Union Medical College, Beijing 100050, China; mayuanyuancircle@163.com

**Keywords:** cathepsin K inhibitors, piperidamide-3-carboxamide derivatives, synthesis, anti-osteoporosis

## Abstract

A series of novel piperidamide-3-carboxamide derivatives were synthesized and evaluated for their inhibitory activities against cathepsin K. Among these derivatives, compound **H-9** exhibited the most potent inhibition, with an IC_50_ value of 0.08 µM. Molecular docking studies revealed that **H-9** formed several hydrogen bonds and hydrophobic interactions with key active-site residues of cathepsin K. In vitro, **H-9** demonstrated anti-bone resorption effects that were comparable to those of MIV-711, a cathepsin K inhibitor currently in phase 2a clinical trials for the treatment of bone metabolic disease. Western blot analysis confirmed that **H-9** effectively downregulated cathepsin K expression in RANKL-reduced RAW264.7 cells. Moreover, in vivo experiments showed that **H-9** increased the bone mineral density of OVX-induced osteoporosis mice. These results suggest that **H-9** is a potent anti-bone resorption agent targeting cathepsin K and warrants further investigation for its potential anti-osteoporosis values.

## 1. Introduction

Osteoporosis is a growing global health issue that significantly affects the elderly population [1], particularly postmenopausal women [2]. It is a systemic skeletal disease characterized by low bone mineral density (BMD) and deterioration of bone architecture, with an increased fracture risk [3]. Currently, it is estimated that more than 200 million people worldwide suffer from osteoporosis, resulting in over 8.9 million fractures annually [4]. Bone health is typically maintained through a balance between the formation of new bone by osteoblasts and the resorption of old or damaged bone by osteoclasts. However, when osteoclast activity exceeds that of osteoblasts, it leads to excessive bone resorption, resulting in osteoporosis. Although several types of anti-osteoporosis drugs, such as calcium supplements [5], bisphosphonates [6], estrogens [7], and denosumab [8], are currently available, the development of novel therapeutic agents could provide additional treatment options for osteoporosis patients.

**Figure 1:**
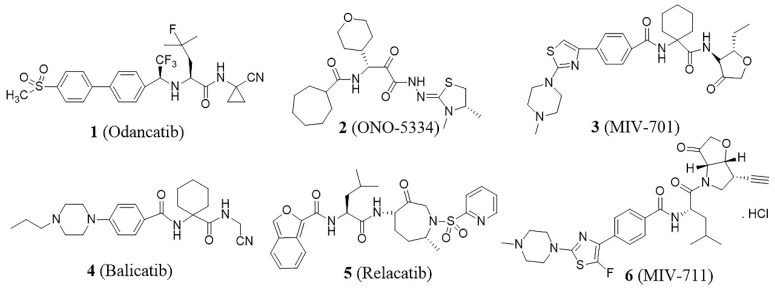
Cat K inhibitors that progressed to the clinic.

Cathepsin K (Cat K), a papain-like cysteine peptidase, is predominantly expressed in osteoclasts and plays a major role in bone resorption. The significance of Cat K in osteoclast function was highlighted by the discovery of mutations in the Cat K gene in patients with pycnodysostosis, a rare recessive disorder [9]. Studies have demonstrated that Cat K possesses unparalleled activity in cleaving the triple helix collagen at multiple sites, a capability unique among mammalian peptidases [10]. The degradation of bone collagen is primarily driven by Cat K activity, making the inhibition of Cat K a promising strategy for combating diseases characterized by excessive bone resorption [11], such as osteoporosis. Over the past decades, considerable efforts have been made to design and develop highly potent and orally bioavailable Cat K inhibitors [12]. Various structural types have been reported, such as oxadiazole [13], chalone, triazine [14], cyclohexanecarboxamide [15], etc. Several of these inhibitors have progressed to clinical trials (Figure 1), including odanacatib (**1**) [16], ONO-5334 (**2**) [17], MIV-701 (**3**), balicatib (**4**) [18], relacatib (**5**) [19], and MIV711 (**6**) [20]. Although some clinical studies have been terminated for various reasons, MIV-711 is currently undergoing a phase 2a clinical trial for the treatment of bone metabolic disorders [21].

## 2. Results and Discussion

### 2.1. Design and Chemistry

**Figure 2:**
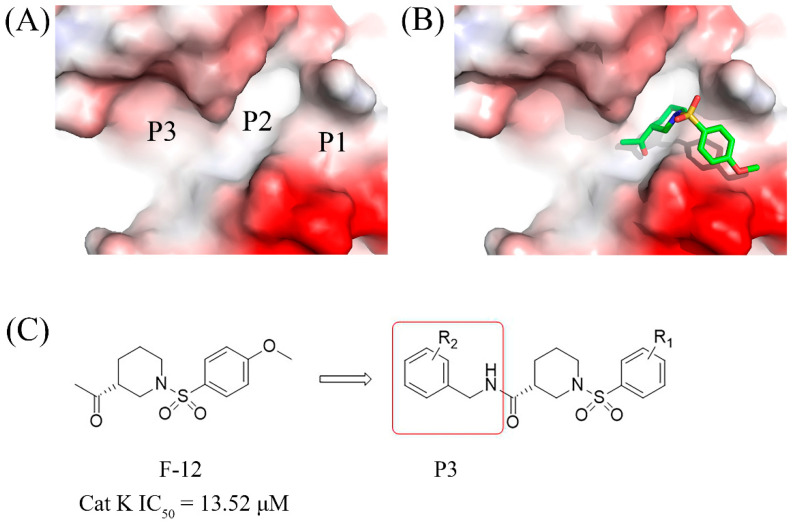
(**A**) The P1, P2, and P3 active pockets of Cat K. (**B**) The binding mode of **F-12** in the active pockets of Cat K. (**C**) The design strategy based on the fragment **F-12**.

In this study, several fragments with Cat K inhibitory activities were identified through virtual screening of our in-house fragment library, notably the sulfonyl piperidine compound **F-12** exhibited the most potent activity, with an IC_50_ value of 13.52 µM. Molecular docking analysis revealed that **F-12** occupied the P1 and P2 pockets of the Cat K active cavity (Figure 2). To enhance its interactions with the P3 pocket, a benzylamine group was introduced to the **F-12** skeleton using a fragment growth strategy. Consequently, a series of novel piperidine-3-carboxamide derivatives were designed, synthesized, and evaluated for their biological activities, to explore potent anti-bone resorption agents with the potential to treat osteoporosis.

**Scheme 1:**
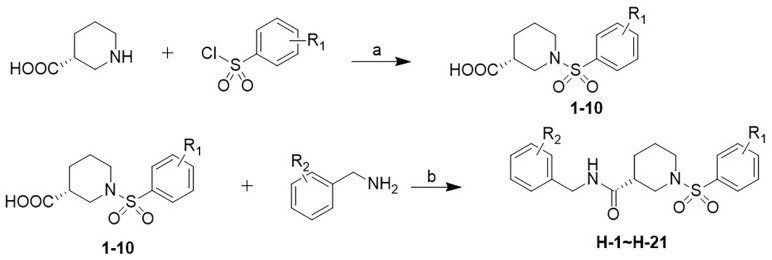
Synthetic route for target compounds **H-1**~**H-21**. Reagents and conditions: (a) NaOH, THF, 4 h, rt; (b) EDCI, DMAP, DCM, 2 h, rt.

The synthesis route for the target compounds **H-1**~**H-21** is illustrated in Scheme 1. The commercially available (*R*)-3-piperidinic acid and corresponding benzene sulfonyl chloride were stirred in the presence of sodium hydroxide in THF to afford intermediates **1**–**10**. Subsequently, the target compounds **H-1**~**H-21** were successfully obtained via an esterification reaction of **1**–**10** with the corresponding benzylamine in CH_2_Cl_2_, catalyzed by EDCI and DMAP.

### 2.2. In Vitro Enzymatic Assays and Preliminary Structure–Activity Relationships

The Cat K inhibitory activities of the target compounds were evaluated, with the bioactivity data summarized in Table 1, using MIV-711 as the positive control. To our delight, most compounds of this class showed moderate-to-strong inhibitory activity. Compared to the lead compound **F-12**, all target compounds exhibited significantly enhanced Cat K inhibitory activity, validating the design strategy of introducing a benzylamine group to enhance interactions with the P3 pocket of Cat K.

**Figure 3:**
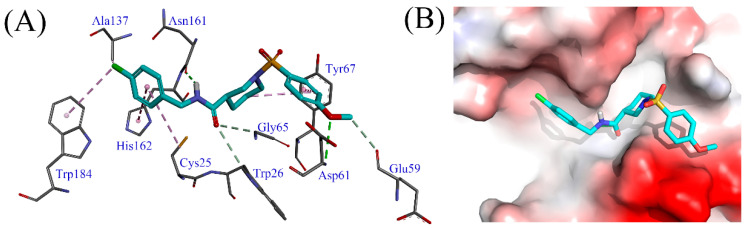
(**A**) The interactions of compound **H-9** with Cat K. (**B**) The binding mode of compound **H-9** in the active site of Cat K. Compound **H-19** is represented as stick diagrams with carbon atoms shown in cyan.

Generally, electron-withdrawing groups at the R_1_ position demonstrated higher potency than electron-donating groups (**H-1**~**H-6**, **H-7**~**H-12**). Among these, the compound with a chloro substituent (**H-7**) showed slightly greater activity compared to the one with a bromo substituent (**H-10**). Additionally, the 4-chloro substitution (**H-7**) appeared more favorable for activity compared to the 2-chloro or 3-chloro substitutions (**H-8**, **H-9**). This enhanced activity is possibly due to the 4-chloro group facilitating σ-σ and σ-π hydrophobic interactions with Ala137 and Trp184 of Cat K (Figure 3). Notably, the 4-methoxy group at the R_2_ position forms a classical hydrogen bond interaction with Asp61, as well as a non-classical hydrogen bond interaction with Glu59 (Figure 3), which may explain the strong Cat K inhibitory activity of compound **H-9**. Conversely, the presence of an electron-withdrawing group at the R_2_ position appears to hinder the activity improvement of the compound (**H-16**).

Molecular docking analyses between Cat K and compound **H-9** demonstrate additional interactions (Figure 3). The phenyl group connected to the sulfonyl moiety and the piperidyl group form π-π and σ-π hydrophobic interactions with Tyr67, respectively. The oxygen atom of the carbonyl group engages in non-classical hydrogen bond interactions with Trp26 and Gly65. Additionally, the amide group acts as a donor, forming a classical hydrogen bond interaction with Asn161. The chlorobenzene ring participates in σ-π and π-π hydrophobic interactions with Cys25 and His162. Overall, the phenyl, acylamino, and piperidyl groups contribute positively to the Cat K inhibitory activity of this class of compounds through their interactions with Cat K. Furthermore, the 4-chloro and 4-methoxy substituents on the benzene rings enhance the activity of the compound by stabilizing these interactions.

### 2.3. In Vitro Anti-Bone Resorption Activity

Type I collagen, the predominant structural protein in bone, constitutes approximately 90% of the bone matrix protein [22]. During bone renewal, type I collagen is degraded, releasing short peptide fragments such as the C-terminal peptide of type I collagen (CTX-I) into the bloodstream. The concentration of CTX-I in the patient’s blood serves as a crucial biomarker for evaluating the degree of osteoporosis. In vitro, bone resorption can be assessed by measuring the concentration of CTX-I using a model in which osteoclasts are cultured on bone slices. In this experiment, the in vitro anti-bone resorption activities of the test compounds were evaluated in RANKL-induced RAW264.7 cells. This evaluation was based on the concentration of CTX-I released into the cell culture medium due to bone degradation, as well as the area of bone resorption pits formed on bone slices.

**Figure 4:**
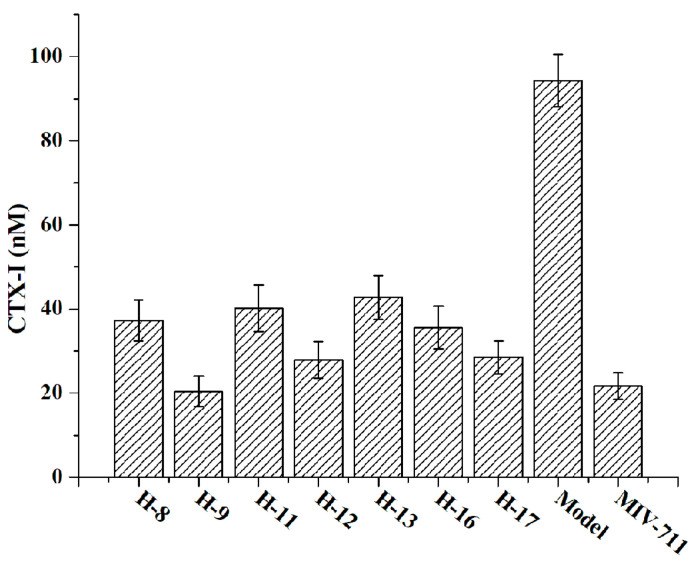
In vitro anti-bone resorption activity evaluated by the concentration of CTX-I.

The concentration of CTX-I in the cell culture medium was detected and quantified by ELISA (Figure 4). Compared to the model group (untreated with drugs), all the treated groups (1 µM) exhibited reduced CTX-I concentration, demonstrating that all the tested compounds (**H-8**, **H-9**, **H-11**, **H-12**, **H-13**, **H-16,** and **H-17**) possess anti-bone resorption activity in vitro. Notably, compound **H-9** (CTX-I, 20.46 ± 3.67 nM) showed anti-bone resorption activity comparable to that of MIV-711 (CTX-I, 21.73 ± 3.18 nM) in RAW264.7 cells, consistent with its enzyme inhibitory activity.

**Figure 5:**
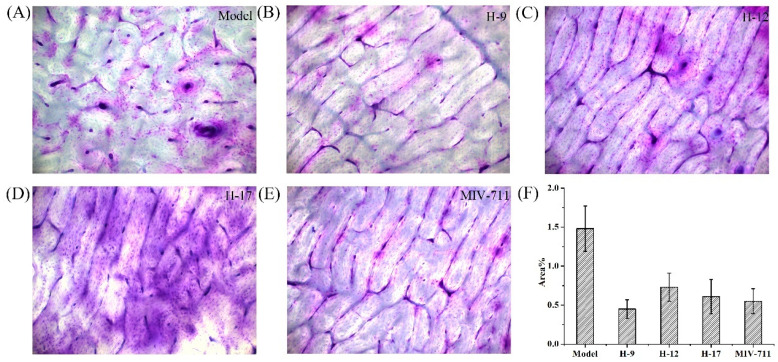
The bone resorption pits of all experimental groups: (**A**) model group, (**B**) H-9, (**C**) H-12, (**D**) H-17, and (**E**) MIV-711; (**F**) Effects of all test compounds on the bone resorption evaluated by the area of bone resorption pits.

Bone resorption pits on bone slices were stained with toluidine blue and analyzed by computer image analysis software. As shown in Figure 5, the areas of bone resorption pits decreased in the drug-treated groups compared with the model group, indicating that the bone resorption process was interrupted by these compounds. Compound **H-9**, **H-12,** and **H-17** exhibited anti-bone resorption activity comparable to that of MIV-711. The reductions in both CTX-I release and bone resorption pit areas confirm the anti-bone resorption efficacy of these compounds in vitro.

### 2.4. Effects on the Expression of Cat K

**Figure 6:**
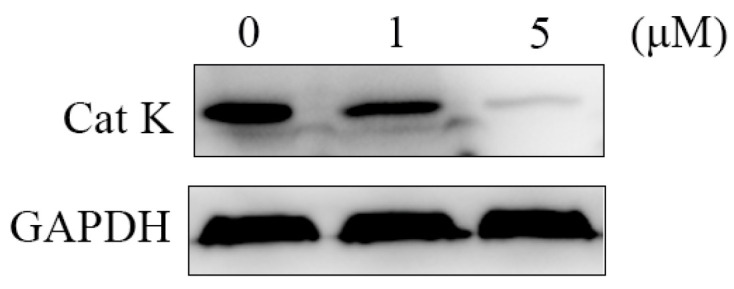
The activity of compound H-9 for regulating Cat K by western blotting in RAW264.7 cells.

To further verify the anti-bone resorption mechanism of the target compounds, a western blotting assay was carried out to assess the effect of compound **H-9** on the expression of Cat K. RANKL-reduced RAW264.7 cells were treated with compound **H-9** at concentrations of 0, 1, and 5 µM for five days, followed by cell lysis. Proteins were extracted and analyzed for Cat K levels. As shown in Figure 6, compound **H-9** caused an obviously dose-dependent decrease in Cat K levels in RAW264.7 cells. These western blotting results, combined with enzyme inhibitory activities and molecular docking analyses, confirm that the anti-bone resorption activity of compound **H-9** is mediated through the inhibition of Cat K activity.

### 2.5. Effects of ***H**-**9*** on OVX-Induced-Osteoporosis Mice

Initially, acute toxicity testing was carried out to establish appropriate dosages. Compound **H-9** was administered orally at a dose of 300 mg/kg/day for 7 days. No mouse deaths were observed during this period, indicating that the LD_50_ value of **H-9** exceeds 300 mg/kg when administered orally.

**Figure 7:**
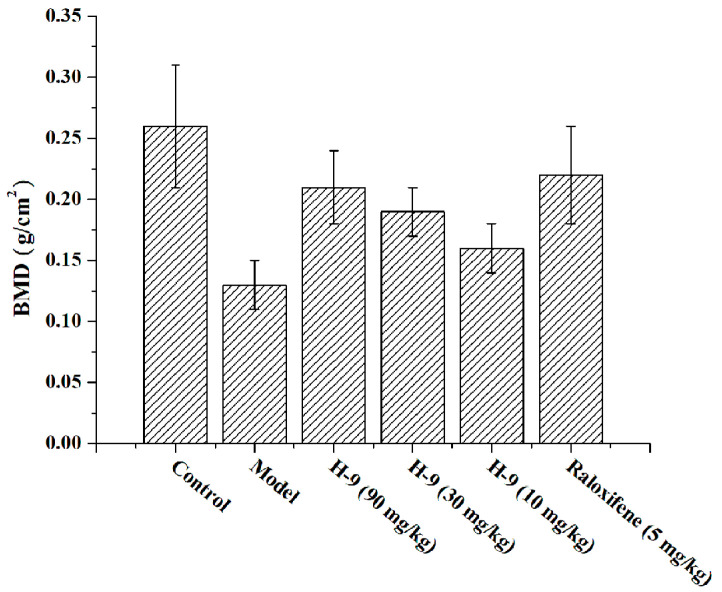
Effects of the compounds on BMD in OVX-induced-osteoporosis mice.

To evaluate the anti-osteoporosis therapeutic potential of compound **H-9**, an ovariectomized (OVX)-induced osteoporosis mouse model was established and employed. The study included six experimental groups: a blank control group, an untreated model group, three **H-9** treatment groups (doses of 90 mg/kg, 30 mg/kg, and 10 mg/kg), and a positive control group treated with raloxifene (5 mg/kg), with 10 mice in each group. As depicted in Figure 7, the BMD of the untreated model group was significantly lower than that of the blank control group, confirming the successful establishment of the osteoporosis model. In contrast, all **H-9** treatment groups and the raloxifene group showed significant increases in BMD compared to the untreated model group, indicating positive effects in combating osteoporosis. The effectiveness of **H-9** demonstrated a dose-dependent relationship in its anti-osteoporosis activity. Notably, the efficacy of **H-9** at 90 mg/kg was comparable to that of the positive control (5 mg/kg). In conclusion, **H-9** exhibited promising therapeutic effects in the OVX-induced osteoporosis mouse model.

## 3. Experimental Section

### 3.1. General Procedures

Each melting points (m.p.) was determined using an MP90 automated melting point instrument (Mettler Toledo, Zurich, Switzerland). The HPLC method was applied for purity assessment on a Waters e2695 (Milford, CO, USA). Column: Supersil ODS2 (4.6 mm × 250 mm, 5 μm); flow-rate: 1.0 mL/min; wavelength: 254 nm; column temperature: 30 °C; mobile phase: acetonitrile (80%) and H_2_O (20%). Mass spectra were acquired using an Agilent 6210 ESI/TOF mass spectrometer (Santa Clara, CA, USA). ^1^H and ^13^C NMR spectra were recorded on a Bruker BioSpin GmbH spectrometer (Billica, MA, USA) at 400 MHz. Column chromatography was carried out on silica gel (200–300 mesh) from Qingdao Ocean Chemical (Qingdao, China). Unless otherwise specified, all reagents were obtained from commercial suppliers.

### 3.2. General Procedure for Preparation of Intermediates (***1***–***10***)

A mixture of 3-piperidinic acid (1.0 g, 7.7 mmol), the corresponding benzene sulfonyl chloride (9.2 mmol), and sodium hydroxide (0.37 g, 9.2 mmol) in THF (20 mL) was stirred at room temperature for 4 h. The reaction progress was monitored by TLC. Upon completion, the solvent was evaporated under reduced pressure. The residue was dissolved in water (20 mL) and neutralized with 4 M HCl. The resulting white precipitate was collected by filtration and purified by recrystallization from ethanol, yielding intermediates **1**–**10**.

(*R*)-1-((4-methoxyphenyl)sulfonyl)piperidine-3-carboxylic acid (**1**): White solid; yield: 64%; ^1^H NMR (400 MHz, DMSO-*d*_6_) *δ* 12.51 (s, 1H), 7.67 (d, *J* = 8.8 Hz, 2H), 7.16 (d, *J* = 8.8 Hz, 2H), 3.85 (s, 3H), 3.49 (d, *J* = 9.1 Hz, 1H), 3.36–3.29 (m, 2H), 2.44–2.31 (m, 2H), 1.80–1.68 (m, 2H), 1.50–1.30 (m, 2H); ^13^C NMR (101 MHz, DMSO-*d*_6_) *δ* 174.24, 163.16, 130.08, 127.32, 115.03, 56.15, 48.00, 46.49, 26.10, 23.83; HRMS calculated for C_13_H_17_NO_5_SNa (M + Na)^+^ 322.0725, found 322.0726.

(*R*)-1-((2-bromophenyl)sulfonyl)piperidine-3-carboxylic acid (**2**): White solid; yield: 70%; ^1^H NMR (400 MHz, DMSO-*d*_6_) *δ* 7.91 (dd, *J* = 7.7, 1.8 Hz, 1H), 7.57 (dd, *J* = 7.8, 1.1 Hz, 1H), 7.33 (td, *J* = 7.6, 1.2 Hz, 1H), 7.22 (td, *J* = 7.6, 1.8 Hz, 1H), 3.46–3.42 (m, 2H), 3.41–3.36 (m, 1H), 3.05–2.78 (m, 2H), 2.29–1.92 (m, 2H), 1.68–1.53 (m, 2H); ^13^C NMR (101 MHz, DMSO-*d*_6_) *δ* 176.52, 147.14, 134.24, 130.69, 129.51, 127.28, 120.04, 67.49, 56.47, 49.34, 46.97, 46.23, 43.95, 29.00, 26.73, 25.59, 25.37, 22.11, 19.02; HRMS calculated for C_12_H_15_NO_4_SBr (M + H)^+^ 347.9905 found 347.9902.

(*R*)-1-((3-bromophenyl)sulfonyl)piperidine-3-carboxylic acid (**3**): White solid; yield: 58%; ^1^H NMR (400 MHz, DMSO-*d*_6_) *δ* 7.94 (dd, *J* = 4.8, 4.1 Hz, 1H), 7.86–7.85 (m, 1H), 7.76 (d, *J* = 7.8 Hz, 1H), 7.62 (t, *J* = 7.9 Hz, 1H), 3.64–3.54 (m, 2H), 3.46–3.35 (m, 1H), 2.46–2.25 (m, 2H), 1.78–1.69 (m, 2H), 1.56–1.34 (m, 2H); ^13^C NMR (101 MHz, DMSO-*d*_6_) *δ* 174.37, 138.16, 136.50, 132.20, 129.95, 126.93, 122.89, 67.48, 56.49, 48.04, 46.48, 26.13, 25.59, 23.96, 19.02; HRMS calculated for C_12_H_15_NO_4_SBr (M + H)^+^ 347.9905, found 347.9901.

(*R*)-1-((4-bromophenyl)sulfonyl)piperidine-3-carboxylic acid (**4**): White solid; yield: 62%; ^1^H NMR (400 MHz, DMSO-*d*_6_) *δ* 7.86 (d, *J* = 8.6 Hz, 2H), 7.67 (d, *J* = 8.6 Hz, 2H), 3.46–3.41 (m, 2H), 3.33–3.30 (m, 1H), 2.58–2.19 (m, 2H), 1.81–1.68 (m, 2H), 1.59–1.32 (m, 2H); ^13^C NMR (101 MHz, DMSO) *δ* 174.12, 135.23, 133.01, 129.84, 127.63, 56.48, 47.85, 46.40, 25.94, 23.84, 19.01; ^13^C NMR (101 MHz, DMSO-*d*_6_) δ 174.37, 138.16, 136.50, 132.20, 129.95, 126.93, 122.89, 67.48, 56.49, 48.04, 46.48, 26.13, 25.59, 23.96, 19.02. HRMS calculated for C_12_H_15_NO_4_SBr (M + H)^+^ 347.9905, found 347.9901.

(*R*)-1-((2-chlorophenyl)sulfonyl)piperidine-3-carboxylic acid (**5**): White solid; yield: 71%; ^1^H NMR (400 MHz, DMSO-*d*_6_) *δ* 7.98 (dd, *J* = 7.8, 1.2 Hz, 1H), 7.71–7.65 (m, 2H), 7.58–7.54 (m, 1H), 3.50–3.45 (m, 1H), 3.43–3.37 (m, 2H), 2.94–2.75 (m, 2H), 1.91–1.69 (m, 2H), 1.57–1.34 (m, 2H); ^13^C NMR (101 MHz, DMSO-*d*_6_) *δ* 174.15, 136.04, 134.93, 132.76, 132.00, 131.38, 128.29, 47.44, 45.93, 26.24, 24.35; HRMS calculated for C_12_H_15_NO_4_SCl (M + H)^+^ 304.0410, found 304.0412.

(*R*)-1-((3-chlorophenyl)sulfonyl)piperidine-3-carboxylic acid (**6**): White solid; yield: 58%; ^1^H NMR (400 MHz, DMSO-*d*_6_) *δ* 7.83– 7.80 (m, 1H), 7.75–7.67 (m, 3H), 3.65–3.51 (m, 2H), 3.45–3.41 (m, 1H), 2.37–2.24 (m, 2H), 1.77–1.69 (m, 2H), 1.57–1.36 (m, 2H); ^13^C NMR (101 MHz, DMSO-*d*_6_) *δ* 174.13, 138.02, 134.62, 133.64, 131.99, 127.23, 126.59, 67.48, 56.49, 47.83, 46.56, 46.45, 25.92, 25.59, 23.84, 19.01; HRMS calculated for C_12_H_15_NO_4_SCl (M + H)^+^ 304.0410, found 304.0406.

(*R*)-1-((4-chlorophenyl)sulfonyl)piperidine-3-carboxylic acid (**7**): White solid; yield: 66%; ^1^H NMR (400 MHz, DMSO-*d*_6_) *δ* 7.75 (d, *J* = 8.9 Hz, 2H), 7.72 (d, *J* = 8.9 Hz, 2H), 3.61–3.41 (m, 2H), 3.36–3.33 (m, 1H), 2.53–2.37 (m, 2H), 1.82–1.68 (m, 2H), 1.53–1.31 (m, 2H); ^13^C NMR (101 MHz, DMSO-*d*_6_) *δ* 174.35, 138.57, 134.86, 130.05, 129.77, 56.48, 48.05, 46.44, 26.14, 23.95, 19.01; HRMS calculated for C_12_H_15_NO_4_SCl (M + H)^+^ 304.0410, found 304.0406.

(*R*)-1-(o-tolylsulfonyl)piperidine-3-carboxylic acid (**8**): White solid; yield: 54%; ^1^H NMR (400 MHz, DMSO-*d*_6_) *δ* 7.81–7.53 (m, 2H), 7.44 (dd, *J* = 9.9, 5.3 Hz, 2H), 3.60–3.50 (m, 2H), 3.39–3.31 (m, 1H), 2.55 (s, 3H), 2.41–2.34 (m, 2H), 1.77–1.69 (m, 2H), 1.53–1.45 (m, 2H); ^13^C NMR (101 MHz, DMSO-*d*_6_) *δ* 174.33, 137.75, 133.40, 130.35, 130.00, 127.91, 126.91, 47.17, 45.54, 26.33, 24.18, 21.47, 20.60; HRMS calculated for C_13_H_18_NO_4_S (M + H)^+^ 284.0957, found 284.0954.

(*R*)-1-(m-tolylsulfonyl)piperidine-3-carboxylic acid (**9**): White solid; yield: 59%; ^1^H NMR (400 MHz, DMSO-*d*_6_) *δ* 7.54 (d, *J* = 6.4 Hz, 4H), 3.64–3.50 (m, 2H), 3.32 (m, 1H), 2.41 (s, 3H), 2.30–2.16 (m, 2H), 1.80–1.68 (m, 2H), 1.51–1.32 (m, 2H); ^13^C NMR (101 MHz, DMSO-*d*_6_) *δ* 174.26, 139.71, 135.85, 134.25, 129.71, 127.93, 125.05, 48.00, 46.52, 26.09, 23.87, 21.29; HRMS calculated for C_13_H_18_NO_4_S (M + H)^+^ 284.0957, found 284.0953.

(*R*)-1-tosylpiperidine-3-carboxylic acid (**10**): White solid; yield: 67%; ^1^H NMR (400 MHz, DMSO-*d*_6_) *δ* 7.62 (d, *J* = 8.2 Hz, 2H), 7.45 (d, *J* = 8.1 Hz, 2H), 3.61–3.41 (m, 2H), 3.34–3.31 (m, 1H), 2.40 (s, 3H), 2.36–2.11 (m, 2H), 1.80–1.67 (m, 2H), 1.50–1.28 (m, 2H); ^13^C NMR (101 MHz, DMSO-*d*_6_) *δ* 174.35, 144.00, 132.94, 130.33, 127.89, 56.49, 48.09, 46.51, 26.20, 23.91, 21.47, 19.01; HRMS calculated for C_13_H_18_NO_4_S (M + H)^+^ 284.0957, found 284.0948.

### 3.3. General Procedure for Preparation of Target Compounds (***H-1***~***H-21***)

A stirred solution of the appropriate intermediate of **1**–**10** (1.12 mmol) and the corresponding benzylamine (1.12 mmol) in 10 mL anhydrous CH_2_Cl_2_ was prepared. EDCI (1.12 mmol) and DMAP (1.12 mmol) were then added successively. The reaction mixture was stirred for 2 h, and the reaction completion was confirmed by TLC analysis. The mixture was washed with 4 M HCl and saturated sodium chloride solution. The organic phase was separated, dried over anhydrous MgSO_4_, and evaporated. The residue was purified by column chromatography using a petroleum ether/ethyl acetate gradient (20:1–10:1) to afford the target compounds **H-1**~**H-21**. The ^1^H NMR spectra, ^13^C NMR spectra, and mass spectra of compounds H-1~H-21 can be found in the Supplementary Materials.

(*R*)-1-((4-methoxyphenyl)sulfonyl)-N-(2-methylbenzyl)piperidine-3-carboxamide (**H-1**): White solid; yield: 51%; purity: 96%; m.p.: 170.4–171.4 °C; ^1^H NMR (400 MHz, DMSO-*d*_6_) *δ* 8.36 (t, *J* = 5.3 Hz, 1H), 7.67 (d, *J* = 8.8 Hz, 2H), 7.17 (d, *J* = 7.9 Hz, 6H), 4.28–4.17 (m, 2H), 3.86 (s, 3H), 3.64–3.56 (m, 2H), 2.24 (s, 3H), 2.20–2.08 (m, 2H), 1.75 (t, *J* = 13.7 Hz, 2H), 1.50–1.23 (m, 2H); ^13^C NMR (101 MHz, DMSO-*d*_6_) *δ* 172.40, 163.19, 137.26, 136.11, 130.38, 130.10, 128.00, 127.36, 127.17, 126.22, 115.03, 56.15, 48.79, 46.51, 42.06, 27.18, 24.19, 19.04; HRMS calculated for C_21_H_27_N_2_O_4_S (M + H)^+^ 403.1692, found 403.1693.

(*R*)-1-((4-methoxyphenyl)sulfonyl)-N-(3-methylbenzyl)piperidine-3-carboxamide (**H-2**): White solid; yield: 63%; purity: 98%; m.p.: 189.8–190.1 °C; ^1^H NMR (400 MHz, DMSO-*d*_6_) *δ* 8.47 (t, *J* = 5.5 Hz, 1H), 7.67 (d, *J* = 8.7 Hz, 2H), 7.19 (dd, *J* = 18.8, 8.1 Hz, 3H), 7.03 (t, *J* = 10.5 Hz, 3H), 4.21 (d, *J* = 3.4 Hz, 2H), 3.86 (s, 3H), 3.86–3.55 (m, 2H), 2.46 (m, 1H), 2.28 (s, 3H), 2.23–2.09 (m, 2H), 1.75 (t, *J* = 12.7 Hz, 2H), 1.52–1.23 (m, 2H); ^13^C NMR (101 MHz, DMSO-*d*_6_) *δ* 172.49, 163.20, 139.70, 137.82, 130.11, 128.69, 128.21, 127.86, 127.18, 124.66, 115.03, 56.15, 48.80, 46.52, 42.35, 42.10, 27.09, 24.17, 21.48; HRMS calculated for C_21_H_27_N_2_O_4_S (M + H)^+^ 403.1692, found 403.1689.

(*R*)-1-((4-methoxyphenyl)sulfonyl)-N-(4-methylbenzyl)piperidine-3-carboxamide (**H-3**): White solid; yield: 58%; purity: 97%; m.p.: 182.3–182.7 °C; ^1^H NMR (400 MHz, DMSO-*d*_6_) *δ* 8.45 (s, 1H), 7.67 (d, *J* = 8.8 Hz, 2H), 7.18–7.10 (m, 6H), 4.23–4.14 (m, 2H), 3.86 (s, 3H), 3.63–3.55 (m, 2H), 2.47–2.44 (m, 1H), 2.27 (s, 3H), 2.22–2.07 (m, 2H), 1.74 (t, *J* = 12.2 Hz, 2H), 1.48–1.23 (m, 2H); ^13^C NMR (101 MHz, DMSO-*d*_6_) *δ* 172.44, 163.18, 136.76, 136.26, 130.10, 129.30, 127.56, 127.18, 115.02, 56.15, 48.77, 46.50, 42.12, 27.12, 24.17, 21.11; HRMS calculated for C_21_H_27_N_2_O_4_S (M + H)^+^ 403.1692, found 403.1694.

(*R*)-N-(2-methoxybenzyl)-1-((4-methoxyphenyl)sulfonyl)piperidine-3-carboxamide (**H-4**): White solid; yield: 49%; purity: 96%; m.p.: 143.7–144.2 °C; ^1^H NMR (400 MHz, DMSO-*d*_6_) *δ* 8.30 (t, *J* = 5.4 Hz, 1H), 7.67 (d, *J* = 8.7 Hz, 2H), 7.25–7.12 (m, 4H), 6.98–6.89 (m, 2H), 4.22 (d, *J* = 2.0 Hz, 2H), 3.85 (s, 3H), 3.79 (s, 3H), 3.64–3.56 (m, 2H), 2.55–2.53 (m, 1H), 2.24–2.09 (m, 2H), 1.80–1.72 (m, 2H), 1.49–1.25 (m, 2H); ^13^C NMR (101 MHz, DMSO-*d*_6_) *δ* 172.59, 163.18, 157.12, 130.10, 128.51, 128.03, 127.18, 127.07, 120.59, 115.02, 110.92, 56.13, 55.74, 48.81, 46.52, 42.09, 37.52, 27.17, 24.20; HRMS calculated for C_21_H_27_N_2_O_5_S (M + H)^+^ 419.1641, found 419.1644.

(*R*)-N-(3-methoxybenzyl)-1-((4-methoxyphenyl)sulfonyl)piperidine-3-carboxamide (**H-5**): White solid; yield: 61%; purity: 99%; m.p.: 130.2–131.2 °C; ^1^H NMR (400 MHz, DMSO-*d*_6_) *δ* 8.50 (t, *J* = 5.7 Hz, 1H), 7.67 (d, *J* = 8.8 Hz, 2H), 7.25–7.15 (m, 3H), 6.80 (d, *J* = 7.9 Hz, 3H), 4.28–4.18 (m, 2H), 3.86 (s, 3H), 3.74 (s, 3H), 3.74–3.56 (m, 2H), 2.23–2.08 (m, 2H), 1.76 (t, *J* = 14.8 Hz, 2H), 1.52–1.25 (m, 2H); ^13^C NMR (101 MHz, DMSO-*d*_6_) *δ* 172.54, 163.18, 159.77, 141.43, 130.09, 129.83, 127.19, 119.66, 115.02, 113.06, 112.64, 56.13, 55.40, 48.79, 46.51, 42.29, 42.14, 27.10, 24.18; HRMS calculated for C_21_H_27_N_2_O_5_S (M + H)^+^ 419.1641, found 419.1639.

(*R*)-N-(4-methoxybenzyl)-1-((4-methoxyphenyl)sulfonyl)piperidine-3-carboxamide (**H-6**): White solid; yield: 56%; purity: 96%; m.p.: 139.4–140.9 °C; ^1^H NMR (400 MHz, DMSO-*d*_6_) *δ* 8.43 (t, *J* = 5.6 Hz, 1H), 7.67 (d, *J* = 8.8 Hz, 2H), 7.17–7.14 (m, 4H), 6.88 (d, *J* = 8.5 Hz, 2H), 4.19–4.16 (m, 2H), 3.86 (s, 3H), 3.73 (s, 3H), 3.73–3.56 (m, 2H), 2.46–2.44 (m, 1H), 2.22–2.07 (m, 2H), 1.74 (t, *J* = 12.0 Hz, 2H), 1.51–1.23 (m, 2H); ^13^C NMR (101 MHz, DMSO-*d*_6_) *δ* 172.39, 163.17, 158.66, 131.72, 130.10, 128.90, 127.18, 115.01, 114.17, 56.13, 55.50, 48.77, 46.50, 42.12, 41.84, 27.13, 24.17; HRMS calculated for C_21_H_27_N_2_O_5_S (M + H)^+^ 419.1641, found 419.1638.

(*R*)-N-(2-chlorobenzyl)-1-((4-methoxyphenyl)sulfonyl)piperidine-3-carboxamide (**H-7**): White solid; yield: 46%; purity: 95%; m.p.: 192.3–193.6 °C; ^1^H NMR (400 MHz, DMSO-*d*_6_) *δ* 8.55 (t, *J* = 5.7 Hz, 1H), 7.67 (d, *J* = 8.8 Hz, 2H), 7.37–7.28 (m, 3H), 7.20–7.15 (m, 3H), 4.31–4.21 (m, 2H), 3.85 (s, 3H), 3.66–3.56 (m, 2H), 2.23–2.08 (m, 2H), 1.76 (t, *J* = 14.8 Hz, 2H), 1.52–1.22 (m, 2H); ^13^C NMR (101 MHz, DMSO-*d*_6_) *δ* 172.68, 163.18, 142.47, 133.45, 130.64, 130.09, 127.34, 127.17, 126.20, 115.01, 56.14, 48.74, 46.50, 42.07, 41.87, 27.04, 24.16; HRMS calculated for C_20_H_24_ClN_2_O_4_S (M + H)^+^ 423.1145, found 423.1146.

(*R*)-N-(3-chlorobenzyl)-1-((4-methoxyphenyl)sulfonyl)piperidine-3-carboxamide (**H-8**): White solid; yield: 67%; purity: 93%; m.p.: 146.1–148.7 °C; ^1^H NMR (400 MHz, DMSO-*d*_6_) *δ* 8.52 (t, *J* = 5.5 Hz, 1H), 7.67 (d, *J* = 8.8 Hz, 2H), 7.43 (d, *J* = 7.4 Hz, 1H), 7.35–7.27 (m, 3H), 7.16 (d, *J* = 8.8 Hz, 2H), 4.37–4.26 (m, 2H), 3.85 (s, 3H), 3.66–3.55 (m, 2H), 2.56 (d, *J* = 11.2 Hz, 1H), 2.25–2.10 (m, 2H), 1.82–1.73 (m, 2H), 1.34–1.22 (m, 2H); ^13^C NMR (101 MHz, DMSO-*d*_6_) *δ* 172.72, 163.19, 136.61, 132.56, 130.10, 129.59, 129.26, 129.10, 127.65, 127.17, 115.02, 56.14, 48.72, 46.52, 42.02, 27.12, 24.17; HRMS calculated for C_20_H_24_ClN_2_O_4_S (M + H)^+^ 423.1145, found 423.1144.

(*R*)-N-(4-chlorobenzyl)-1-((4-methoxyphenyl)sulfonyl)piperidine-3-carboxamide (**H-9**): White solid; yield: 52%; purity: 98%; m.p.: 139.3–140.5 °C; ^1^H NMR (400 MHz, DMSO-*d*_6_) *δ* 8.54 (t, *J* = 5.7 Hz, 1H), 7.67 (d, *J* = 8.8 Hz, 2H), 7.37 (d, *J* = 8.3 Hz, 2H), 7.25 (d, *J* = 8.3 Hz, 2H), 7.16 (d, *J* = 8.8 Hz, 2H), 4.29–4.18 (m, 2H), 3.85 (s, 3H), 3.65–3.55 (m, 2H), 2.23–2.08 (m, 2H), 1.75 (t, *J* = 14.8 Hz, 2H), 1.52–1.22 (m, 2H); ^13^C NMR (101 MHz, DMSO-*d*_6_) *δ* 172.62, 163.18, 138.91, 131.78, 130.10, 129.41, 128.69, 127.19, 115.01, 56.13, 48.72, 46.50, 42.08, 41.75, 27.10, 24.16; HRMS calculated for C_20_H_24_ClN_2_O_4_S (M + H)^+^ 423.1145, found 423.1147.

(*R*)-N-(2-bromobenzyl)-1-((4-methoxyphenyl)sulfonyl)piperidine-3-carboxamide (**H-10**): White solid; yield: 55%; purity: 96%; m.p.: 153.7–154.5 °C; ^1^H NMR (400 MHz, DMSO-*d*_6_) *δ* 8.53 (t, *J* = 5.5 Hz, 1H), 7.67 (d, *J* = 8.8 Hz, 2H), 7.60 (d, *J* = 7.8 Hz, 1H), 7.38 (t, *J* = 7.3 Hz, 1H), 7.29 (d, *J* = 7.1 Hz, 1H), 7.23–7.15 (m, 3H), 4.29–4.27 (m, 2H), 3.86 (s, 3H), 3.67–3.55 (m, 2H), 2.57–2.52 (m, 1H), 2.25–2.10 (m, 2H), 1.78 (dd, *J* = 25.6, 12.9 Hz, 2H), 1.5 –1.25 (m, 2H); ^13^C NMR (101 MHz, DMSO-*d*_6_) *δ* 172.72, 163.19, 138.11, 132.85, 130.11, 129.40, 129.26, 128.23, 122.89, 115.03, 56.15, 48.73, 46.52, 42.91, 42.01, 27.12, 24.17; HRMS calculated for C_20_H_24_BrN_2_O_4_S (M + H)^+^ 467.0640, found 467.0642.

(*R*)-N-(3-bromobenzyl)-1-((4-methoxyphenyl)sulfonyl)piperidine-3-carboxamide (**H-11**): White solid; yield: 60%; purity: 98%; m.p.: 124.7–126.5 °C; ^1^H NMR (400 MHz, DMSO-*d*_6_) *δ* 8.55 (t, *J* = 5.7 Hz, 1H), 7.67 (d, *J* = 8.8 Hz, 2H), 7.45–7.41 (m, 2H), 7.31–7.22 (m, 2H), 7.16 (d, *J* = 8.8 Hz, 2H), 4.25–4.24 (m, 2H), 3.85 (s, 3H), 3.65–3.56 (m, 2H), 2.21–2.08 (m, 2H), 1.75 (t, *J* = 13.5 Hz, 2H), 1.32–1.23 (m, 2H); ^13^C NMR (101 MHz, DMSO-*d*_6_) *δ* 172.68, 163.20, 142.75, 130.98, 130.24, 130.11, 130.09, 127.17, 126.62, 122.10, 115.04, 56.16, 48.75, 46.51, 42.07, 41.81, 27.02, 24.15; HRMS calculated for C_20_H_24_BrN_2_O_4_S (M + H)^+^ 467.0640, found 467.0639.

(*R*)-N-(4-bromobenzyl)-1-((4-methoxyphenyl)sulfonyl)piperidine-3-carboxamide (**H-12**): White solid; yield: 53%; purity: 99%; m.p.: 148.1–149.3 °C; ^1^H NMR (400 MHz, DMSO-*d*_6_) *δ* 8.54 (t, *J* = 5.7 Hz, 1H), 7.67 (d, *J* = 8.7 Hz, 2H), 7.51 (d, *J* = 8.2 Hz, 2H), 7.18 (dd, *J* = 11.0, 8.7 Hz, 4H), 4.27–4.16 (m, 2H), 3.85 (s, 3H), 3.64–3.55 (m, 2H), 2.48–2.45 (m, 1H), 2.23–2.08 (m, 2H), 1.75 (t, *J* = 14.3 Hz, 2H), 1.51–1.23 (m, 2H); ^13^C NMR (101 MHz, DMSO-*d*_6_) *δ* 172.62, 163.18, 139.35, 131.61, 130.10, 129.79, 127.19, 120.22, 115.02, 56.14, 48.71, 46.50, 42.07, 41.81, 40.63, 40.42, 27.09, 24.16; HRMS calculated for C_20_H_24_BrN_2_O_4_S (M + H)^+^ 467.0640, found 467.0638.

(*R*)-1-((2-bromophenyl)sulfonyl)-N-(4-methoxybenzyl)piperidine-3-carboxamide (**H-13**): White solid; yield: 44%; purity: 94%; m.p.: 189.5–190.5 °C; ^1^H NMR (400 MHz, DMSO-*d*_6_) *δ* 8.43 (t, *J* = 5.8 Hz, 1H), 8.00 (dd, *J* = 7.6, 1.9 Hz, 1H), 7.88 (dd, *J* = 7.6, 1.5 Hz, 1H), 7.63–7.55 (m, 2H), 7.14 (d, *J* = 8.6 Hz, 2H), 6.89–6.86 (m, 2H), 4.22–4.12 (m, 2H), 3.76–3.62 (m, 2H), 3.72 (s, 3H), 2.81–2.64 (m, 2H), 2.46–2.41 (m, 1H), 1.87–1.71 (m, 2H), 1.49–1.42 (m, 2H); ^13^C NMR (101 MHz, DMSO-*d*_6_) *δ* 172.44, 158.65, 137.75, 136.31, 134.86, 132.11, 131.75, 128.89, 128.77, 119.95, 114.16, 55.51, 48.13, 45.97, 42.44, 41.81, 27.49, 24.65; HRMS calculated for C_20_H_24_N_2_O_4_SBr (M + H)^+^ 467.0640, found 467.0642.

(*R*)-1-((3-bromophenyl)sulfonyl)-N-(4-methoxybenzyl)piperidine-3-carboxamide (**H-14**): White solid; yield: 47%; purity: 94%; m.p.: 203.5–204.2 °C; ^1^H NMR (400 MHz, DMSO-*d*_6_) *δ* 8.43 (t, *J* = 5.8 Hz, 1H), 7.96–7.94 (m, 1H), 7.87 (t, *J* = 1.7 Hz, 1H), 7.76 (d, *J* = 8.1 Hz, 1H), 7.62 (t, *J* = 7.9 Hz, 1H), 7.15 (d, *J* = 8.6 Hz, 2H), 6.90–6.86 (m, 2H), 4.23– 4.12 (m, 2H), 3.72 (s, 3H), 3.68–3.60 (m, 2H), 2.47–2.42 (m, 1H), 2.34–2.21 (m, 2H), 1.78–1.73 (m, 2H), 1.48–1.17 (m, 2H); ^13^C NMR (101 MHz, DMSO-*d*_6_) *δ* 172.25, 158.66, 138.03, 136.54, 132.20, 131.69, 129.97, 128.90, 126.95, 122.91, 114.17, 56.50, 55.51, 48.59, 46.45, 42.10, 41.85, 27.05, 24.22, 19.02; HRMS calculated for C_20_H_24_N_2_O_4_SBr (M + H)^+^ 467.0640, found 467.0635.

(*R*)-1-((4-bromophenyl)sulfonyl)-N-(4-methoxybenzyl)piperidine-3-carboxamide (**H-15**): White solid; yield: 63%; purity: 94%; m.p.: 152.4–154.4 °C; ^1^H NMR (400 MHz, DMSO-*d*_6_) *δ* 8.42 (*t*, *J* = 5.7 Hz, 1H), 7.87 (d, *J* = 8.6 Hz, 2H), 7.67 (d, *J* = 8.6 Hz, 2H), 7.15 (d, *J* = 8.6 Hz, 2H), 6.88 (d, *J* = 8.6 Hz, 2H), 4.22– 4.12 (m, 2H), 3.73 (s, 3H), 3.65–3.57(m, 2H), 2.48–2.43 (m, 1H), 2.31–2.18(m, 2H), 1.78–1.71 (m, 2H), 1.47–1.23 (m, 2H); ^13^C NMR (101 MHz, DMSO-*d*_6_) *δ* 172.26, 158.66, 135.18, 133.00, 131.70, 129.85, 128.91, 127.64, 114.17, 56.50, 55.52, 48.62, 46.40, 42.09, 41.85, 27.05, 24.19, 19.02; HRMS calculated for C_20_H_24_N_2_O_4_SBr (M + H)^+^ 467.0640, found 467.0638.

(*R*)-1-((2-chlorophenyl)sulfonyl)-N-(4-methoxybenzyl)piperidine-3-carboxamide (**H-16**): White solid; yield: 59%; purity: 98%; m.p.: 162.3–164.5 °C; ^1^H NMR (400 MHz, DMSO-*d*_6_) *δ* 8.43 (t, *J* = 5.8 Hz, 1H), 7.97 (dd, *J* = 7.9, 1.3 Hz, 1H), 7.72–7.65 (m, 2H), 7.57 (ddd, *J* = 8.6, 7.0, 1.7 Hz, 1H), 7.14 (d, *J* = 8.6 Hz, 2H), 6.89–6.85 (m, 2H), 4.22–4.12 (m, 2H), 3.67–3.42 (m, 2H), 2.78–2.61(m, 2H), 2.45–2.39 (m, 1H), 1.86–1.72 (m, 2H), 1.48–1.41 (m, 2H); ^13^C NMR (101 MHz, DMSO-*d*_6_) *δ* 172.40, 158.65, 135.99, 134.93, 132.77, 132.00, 131.76, 131.37, 128.88, 128.31, 114.16, 48.17, 46.02, 42.46, 41.81, 27.45, 24.70, 19.02; HRMS calculated for C_20_H_24_N_2_O_4_SCl (M + H)^+^ 423.1145, found 423.1143.

(*R*)-1-((3-chlorophenyl)sulfonyl)-N-(4-methoxybenzyl)piperidine-3-carboxamide (**H-17**): White solid; yield: 55%; purity: 98%; m.p.: 117.1–119.9 °C; ^1^H NMR (400 MHz, DMSO-*d*_6_) *δ* 8.43 (t, *J* = 5.8 Hz, 1H), 7.82 (dt, *J* = 7.1, 1.9 Hz, 1H), 7.76–7.67 (m, 3H), 7.15 (d, *J* = 8.6 Hz, 2H), 6.90–6.86 (m, 2H), 4.23–4.12 (m, 2H), 3.72 (s, 3H), 3.69–3.60 (m, 2H), 2.48–2.43 (m, 1H), 2.34–2.21 (m, 2H), 1.78–1.72 (m, 2H), 1.51–1.28 (m, 2H); ^13^C NMR (101 MHz, DMSO-*d*_6_) *δ* 172.25, 158.67, 137.92, 134.63, 133.65, 131.99, 131.70, 128.90, 127.25, 126.60, 114.17, 56.50, 55.51, 48.60, 46.45, 42.10, 41.85, 27.05, 24.22, 19.02; HRMS calculated for C_20_H_24_N_2_O_4_SCl (M + H)^+^ 423.1145, found 423.1144.

(*R*)-1-((4-chlorophenyl)sulfonyl)-N-(4-methoxybenzyl)piperidine-3-carboxamide (**H-18**): White solid; yield: 60%; purity: 99%; m.p.: 147.0–148.7 °C; ^1^H NMR (400 MHz, DMSO-*d*_6_) *δ* 8.42 (t, *J* = 5.8 Hz, 1H), 7.77–7.71 (m, 4H), 7.15 (d, *J* = 8.6 Hz, 2H), 6.90–6.86 (m, 2H), 4.22–4.12 (m, 2H), 3.73 (s, 3H), 3.66–3.57 (m, 2H), 2.48–2.42 (m, 1H), 2.31–2.17 (m, 2H), 1.78–1.71 (m, 2H), 1.50–1.23 (m, 2H); ^13^C NMR (101 MHz, DMSO-*d***_6_**) *δ* 172.26, 158.66, 138.62, 134.77, 131.70, 130.06, 129.78, 128.91, 114.17, 56.50, 55.51, 48.63, 46.40, 42.09, 41.85, 27.05, 24.19, 19.02; HRMS calculated for C_20_H_24_N_2_O_4_SCl (M + H)^+^ 423.1145, found 423.1144.

(*R*)-N-(4-methoxybenzyl)-1-(o-tolylsulfonyl)piperidine-3-carboxamide (**H-19**): White solid; yield: 50%; purity: 97%; m.p.: 108.3–109.4 °C; ^1^H NMR (400 MHz, DMSO-*d*_6_) *δ* 8.42 (t, *J* = 5.6 Hz, 1H), 7.80– 7.56 (m, 2H), 7.47–7.43 (m, 2H), 7.14 (dd, *J* = 8.8, 2.6 Hz, 2H), 6.89–6.85 (m, 2H), 4.21–4.11 (m, 2H), 3.72 (s, 3H), 3.64–3.54 (m, 2H), 2.55 (s, 3H), 2.46–2.41 (m, 2H), 2.22–2.08 (m, 1H), 1.84–1.71 (m, 2H), 1.48–1.23 (m, 2H); ^13^C NMR (101 MHz, DMSO-*d*_6_) *δ* 172.45, 158.65, 137.65, 136.08, 133.49, 133.41, 131.77, 130.34, 129.95, 128.89, 127.92, 126.94, 114.16, 55.51, 47.85, 45.65, 42.25, 41.82, 27.42, 24.51, 20.66; HRMS calculated for C_21_H_27_N_2_O_4_S (M + H)^+^ 403.1692, found 403.1693.

(*R*)-N-(4-methoxybenzyl)-1-(m-tolylsulfonyl)piperidine-3-carboxamide (**H-20**): White solid; yield: 68%; purity: 98%; m.p.: 152.6–154.2 °C; ^1^H NMR (400 MHz, DMSO-*d*_6_) *δ* 8.43 (t, *J* = 5.8 Hz, 1H), 7.54 (d, *J* = 4.0 Hz, 4H), 7.15 (d, *J* = 8.6 Hz, 2H), 6.90–6.86 (m, 2H), 4.22–4.11 (m, 2H), 3.72 (s, 3H), 3.66–3.58 (m, 2H), 2.46–2.43 (m, 1H), 2.42 (s, 3H), 2.25–2.12 (m, 2H), 1.77–1.72 (m, 2H), 1.51–1.23 (m, 2H); ^13^C NMR (101 MHz, DMSO-*d*_6_) *δ* 172.35, 158.66, 139.70, 135.72, 134.26, 131.72, 129.71, 128.89, 127.93, 125.07, 114.16, 56.50, 55.51, 48.70, 46.51, 42.16, 41.84, 27.12, 24.22, 21.30, 19.01; HRMS calculated for C_21_H_27_N_2_O_4_S (M + H)^+^ 403.1692, found 403.1693.

(*R*)-N-(4-methoxybenzyl)-1-tosylpiperidine-3-carboxamide (**H-21**): White solid; yield: 59%; purity: 98%; m.p.: 162.9–163.7 °C; ^1^H NMR (400 MHz, DMSO-*d*_6_) *δ* 8.43 (t, *J* = 5.8 Hz, 1H), 7.62 (d, *J* = 8.2 Hz, 2H), 7.46 (d, *J* = 8.1 Hz, 2H), 7.15 (d, *J* = 8.6 Hz, 2H), 6.90–6.86 (m, 2H), 4.22–4.12 (m, 2H), 3.72 (s, 3H), 3.64–3.56 (m, 2H), 2.48–2.43 (m, 1H), 2.41 (s, 3H), 2.22–2.08 (m, 2H), 1.77–1.71 (m, 2H), 1.50–1.21 (m, 2H); ^13^C NMR (101 MHz, DMSO-*d*_6_) *δ* 172.37, 158.66, 144.04, 132.78, 131.71, 130.33, 128.90, 127.91, 114.17, 56.50, 55.51, 48.74, 46.49, 42.12, 41.84, 27.13, 24.18, 21.47, 19.01; HRMS calculated for C_21_H_27_N_2_O_4_S (M + H)^+^ 403.1692, found 403.1686.

### 3.4. In Vitro Enzymatic Assays

Compounds were tested at eight concentrations (100–0.001 µM). Recombinant human Cat K (5.5 ng/mL) in reaction buffer (50 µL) containing sodium acetate (50 mM), EDTA (2.5 mM), and DTT (1 mM) was added to a 96-well plate containing the test compounds at pH 6.0. The plate was incubated at 37 °C for 15 min, and the fluorescence (F1) was measured at 355 nm excitation and 460 nm emission using a microplate reader (Wallac 1420 Victor2, Norwalk, CA, USA). Subsequently, Z-Phe-Arg-AMC (20 µL, 10 µM) was added, and the plate was incubated at 37 °C for 1 h. Fluorescence (F2) was measured again at 355 nm excitation and 460 nm emission. The inhibition rate was calculated using the formula: inhibition (%) = [control (F2 − F1) − sample (F2–F1)]/[control (F2 − F1) − blank (F2 − F1)] × 100%. IC_50_ values were calculated using the SPSS 17.0 software.

### 3.5. Molecular Docking

The crystal structure of Cat K (PDB code: 1NLK) was downloaded from the PDB database. For the docking studies, the B chain of 1NLK was selected. Ligands and water molecules were removed from the protein structure, and hydrogen atoms were added. The biopolymer was protonated at pH 7.4, and the Amber7 FF99 force field was applied. Compound **H-9** was minimized following the minimization of Cat K. Default parameters were used for all other settings. The minimized protein and compound **H-9** were then subjected to the docking protocol using the Surflex-Dock Geom mode in Sybyl-X2.0.

### 3.6. In Vitro Anti-Bone Resorption Assay

RAW264.7 cells, obtained from the cell bank of the Chinese Academy of Medical Sciences, were seeded into 96-well plates at a density of 1 × 10^5^ cells per well with bovine femur slices. To induce cell differentiation, 50 ng mL^−1^ of RANKL was added to the RAW264.7 cells. The cells were cultured for 5 days at 37 °C under 5% CO_2_ with the test compounds at a concentration of 1 µM. The culture supernatant was collected to measure the CTX-I concentration using ELISA. The bone slices were fixed with 2.5% glutaraldehyde for 7 min, then washed 3 times with 0.25 mol/L ammonium hydroxide, dehydrated using a series of ethanol solutions (40%, 75%, 80%, 95%, and anhydrous ethanol), and dried naturally. After staining with 1% toluidine blue solution at room temperature for 3–4 min, the bone slices were rinsed with distilled water and analyzed using an optical microscope. Bone resorption areas were calculated using image analysis software (iBright CL1500, Redmond, WA, USA).

### 3.7. Western Blotting Assay

RAW264.7 cells were seeded into 12-well plates at a density of 1 × 10^5^ cells per well, and 50 ng mL^−1^ of RANKL was added to induce cell differentiation. The cells were cultured at 37 °C under 5% CO_2_ and treated with compound **H-9** (0, 1, and 5 µM) for 5 days. The cells were then harvested, lysed, and centrifuged at 12,000× *g* for 10 min. The supernatants were collected and mixed with loading buffer, then heated to 100 °C. Total protein concentrations were determined using a Bio-Rad protein assay (Thermo Fisher Scientific, Hercules, CA, USA). The samples were subjected to SDS-PAGE and transferred to a PVDF membrane. The membrane was blocked with 5% non-fat milk, and then, incubated overnight at 4 °C with primary antibody against Cat K and GAPDH. After incubation with an appropriate secondary antibody conjugated with peroxidase, proteins were visualized using the ECL chemiluminescence system. GAPDH was used as a loading control.

### 3.8. In Vivo Anti-Osteoporosis Experiment

The mice were randomly divided into 2 groups: a blank control group (*n* = 10) and a model group (*n* = 50). After a 7-day acclimation period, the model group mice received a bilateral ovariectomy. Subsequently, the model group was further divided into 5 subgroups (*n* = 10): an untreated model group, three test groups, and a raloxifene group. Ten days after surgery, the three test groups were administered compound **H-9** via intragastric administration at doses of 90 mg/kg, 30 mg/kg, and 10 mg/kg, respectively. Raloxifene was given to the positive control group at a dose of 5 mg/kg. All drugs were administered for 16 weeks, with a 1-day break following every 6 consecutive days of administration. The blank control and untreated model groups received an equivalent volume of saline. At the end of the administration period, the mice were anesthetized with sodium pentobarbital, and the BMD of the thighbone was measured using an OSTEOCORE 3 Bone Density Meter (Thule, Marseille, France).

All animal procedures were performed in accordance with the Guidelines for Care and Use of Laboratory Animals of Jiujiang University and were approved by the Medical Ethics Committee of Jiujiang University (JJU20220057).

## 4. Conclusions

In this study, a library of new piperidamide-3-carboxamide derivatives was designed, synthesized, and evaluated for their biological activity. Preliminary structure–activity relationship studies demonstrated that a 4-electron withdrawing group at the R_1_ position and a 4-electron donating group at the R_2_ position are beneficial to enhancing Cat K’s inhibitory activity. Among the synthesized compounds, **H-9** emerged as the most potent agent, with an IC_50_ value of 0.08 µM. Molecular docking studies revealed that compound **H-9** forms hydrogen bond interactions with Trp26, Glu59, Asp61, Gly65, and Asn161, as well as hydrophobic interactions with Cys25, Trp67, Ala137, His162, and Trp184 of Cat K. Notably, the 4-chloro substituent at the R_1_ position and the 4-methoxy substituent at the R_2_ position significantly contributed to the enhanced Cat K inhibitory activity, as evidenced by enzyme assays and molecular docking results. Additionally, **H-9** exhibited anti-bone resorption potency comparable to that of MIV-711 in vitro. Western blot analysis demonstrated that **H-9** decreased Cat K levels in RAW264.7 cells. In vivo anti-osteoporosis evaluations indicated that **H-9** has positive therapeutic effects in a dose-dependent manner. In conclusion, **H-9** represents a novel scaffold for anti-bone resorption agents targeting Cat K, and further studies are warranted to explore its therapeutic potential.

## Figures and Tables

**Table 1:** The Cat K inhibitory activities of the synthesized compounds.

Compd.	R_1_	R_2_	Cat K IC_50_ (µM) ± SD
**H-1**	2-CH_3_	4-OCH_3_	3.52 ± 0.44
**H-2**	3-CH_3_	4-OCH_3_	4.76 ± 0.71
**H-3**	4-CH_3_	4-OCH_3_	4.39 ± 0.65
**H-4**	2-OCH_3_	4-OCH_3_	2.48 ± 0.37
**H-5**	3-OCH_3_	4-OCH_3_	2.93 ± 0.40
**H-6**	4-OCH_3_	4-OCH_3_	3.64 ± 0.39
**H-7**	2-Cl	4-OCH_3_	1.06 ± 0.18
**H-8**	3-Cl	4-OCH_3_	0.45 ± 0.11
**H-9**	4-Cl	4-OCH_3_	0.08 ± 0.03
**H-10**	2-Br	4-OCH_3_	2.18 ± 0.39
**H-11**	3-Br	4-OCH_3_	0.66 ± 0.20
**H-12**	4-Br	4-OCH_3_	0.22 ± 0.05
**H-13**	4-Br	2-OCH_3_	0.74 ± 0.12
**H-14**	4-Br	3-OCH_3_	0.91 ± 0.15
**H-15**	3-Br	3-OCH_3_	0.85 ± 0.19
**H-16**	4-Cl	4-Cl	2.03 ± 0.43
**H-17**	4-Cl	3-OCH_3_	0.47 ± 0.12
**H-18**	3-Cl	3-OCH_3_	0.78 ± 0.30
**H-19**	4-OCH_3_	2-CH_3_	5.33 ± 0.94
**H-20**	4-OCH_3_	3-CH_3_	3.82 ± 0.91
**H-21**	4-OCH_3_	4-CH_3_	3.65 ± 0.86
**F-12**	-	-	13.52 ± 2.16
**MIV-711**	-	-	0.09 ± 0.02

## Data Availability

The data presented in this study are available on request from the corresponding author.

## Supplementary Materials

The following supporting information can be downloaded at: https://www.mdpi.com/article/10.3390/molecules29174011/s1, The ^1^H NMR spectra, ^13^C NMR spectra, and mass spectra of compounds **H1**–**H21**.
