# Supplementary material for: Synthesis and Biological Evaluation of Novel Piperidine-3-Carboxamide Derivatives as Anti-Osteoporosis Agents Targeting Cathepsin K
Source: Molecules. 2024 Aug 24;29(17):4011. doi: 10.3390/molecules29174011 (PMC11396514; doi:10.3390/molecules29174011)
Supplement: Supplementary file 1 [file molecules-29-04011-s001.zip › molecules-3145112-supplementary.pdf]

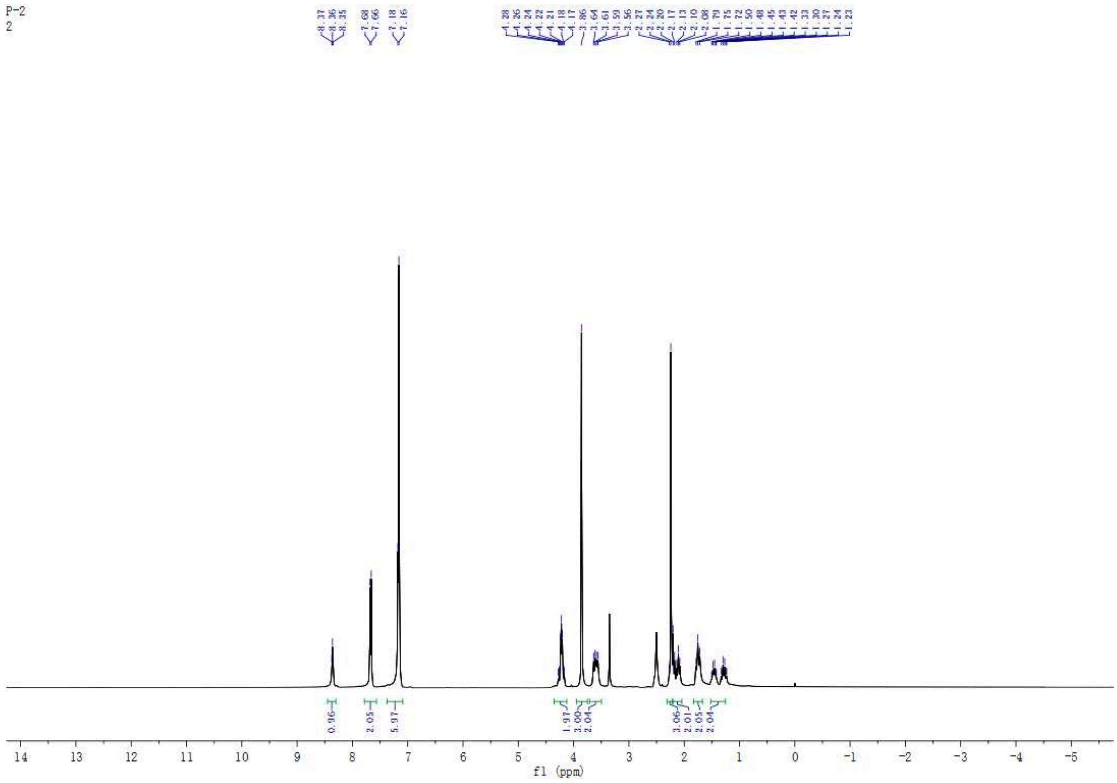

^1H NMR spectrum of compound H-1 (400 MHz, DMSO)

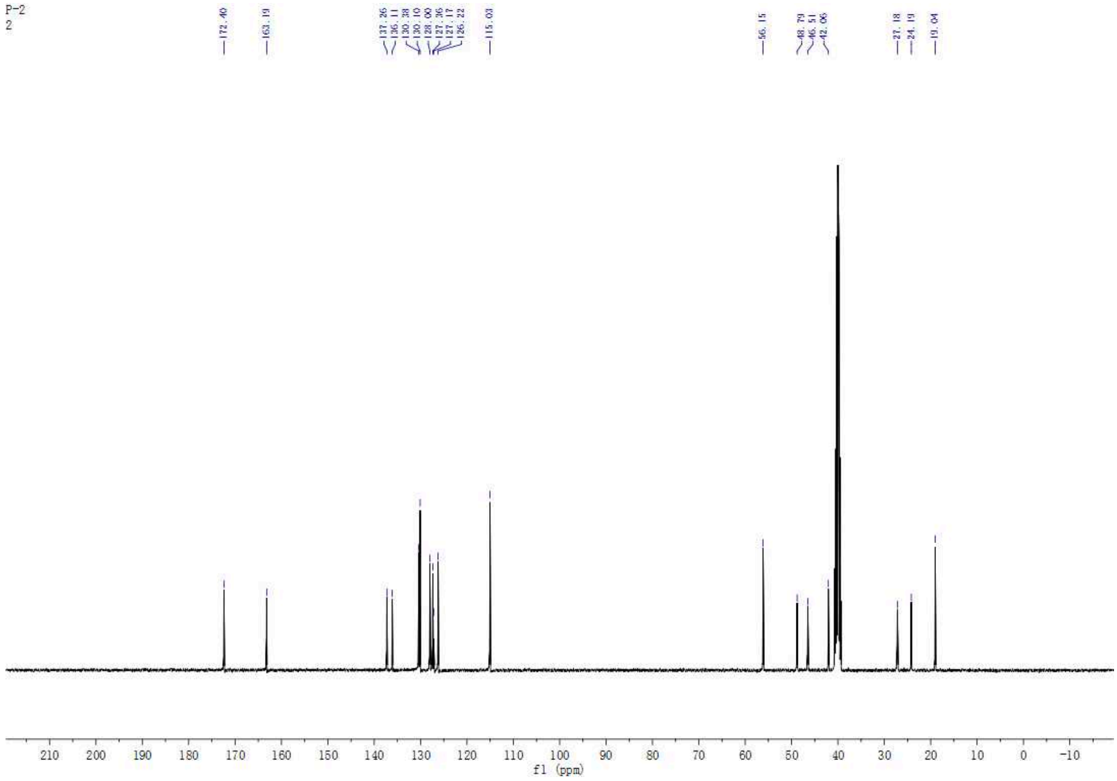

^{13}C NMR spectrum of compound H-1 (101 MHz, DMSO)

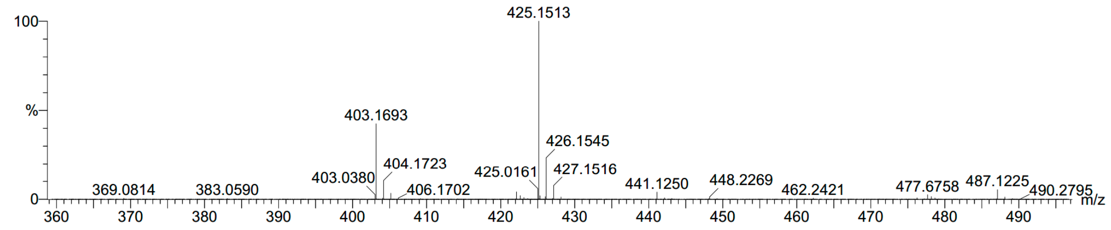

Minimum:				-1.5				
Maximum:		5.0	10.0	50.0				
Mass	Calc. Mass	mDa	PPM	DBE	i-FIT	Norm	Conf(%)	Formula
403.1693	403.1692	0.1	0.2	9.5	657.5	n/a	n/a	C21 H27 N2 O4 S

High resolution mass spectrum of compound H-1

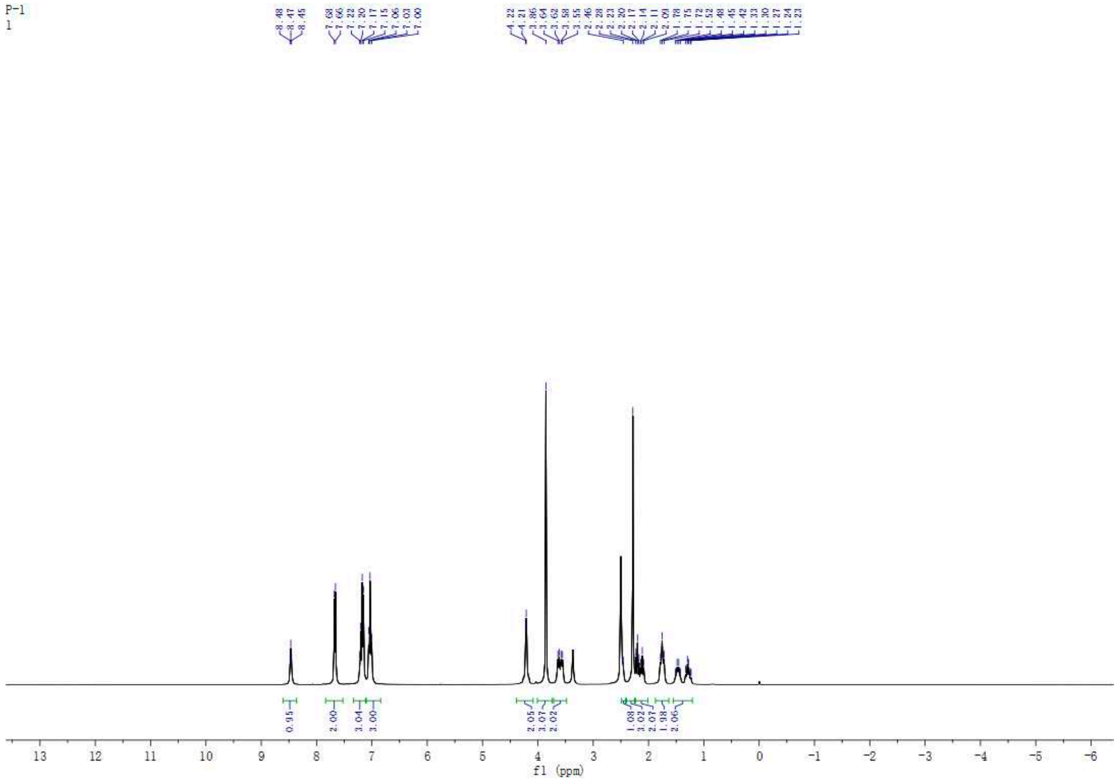

^{1}H NMR spectrum of compound H-2 (400 MHz, DMSO)

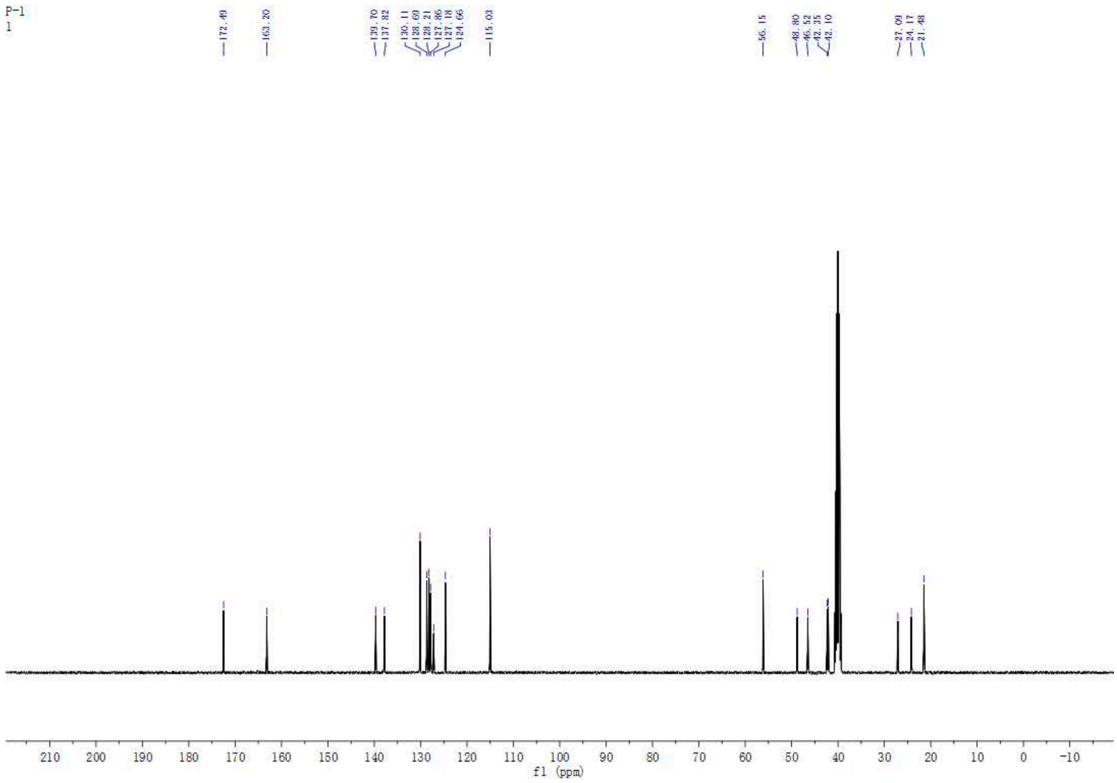

^{13}C NMR spectrum of compound H-2 (101 MHz, DMSO)

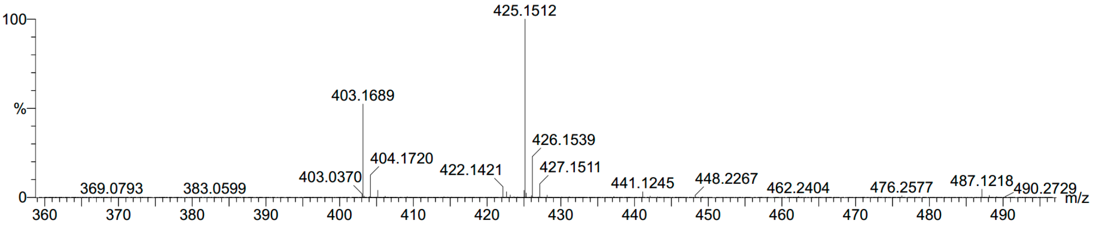

Minimum:				-1.5				
Maximum:		5.0	10.0	50.0				
Mass	Calc. Mass	mDa	PPM	DBE	i-FIT	Norm	Conf(%)	Formula
403.1689	403.1692	-0.3	-0.7	9.5	685.1	n/a	n/a	C21 H27 N2 O4 S

High resolution mass spectrum of compound H-2

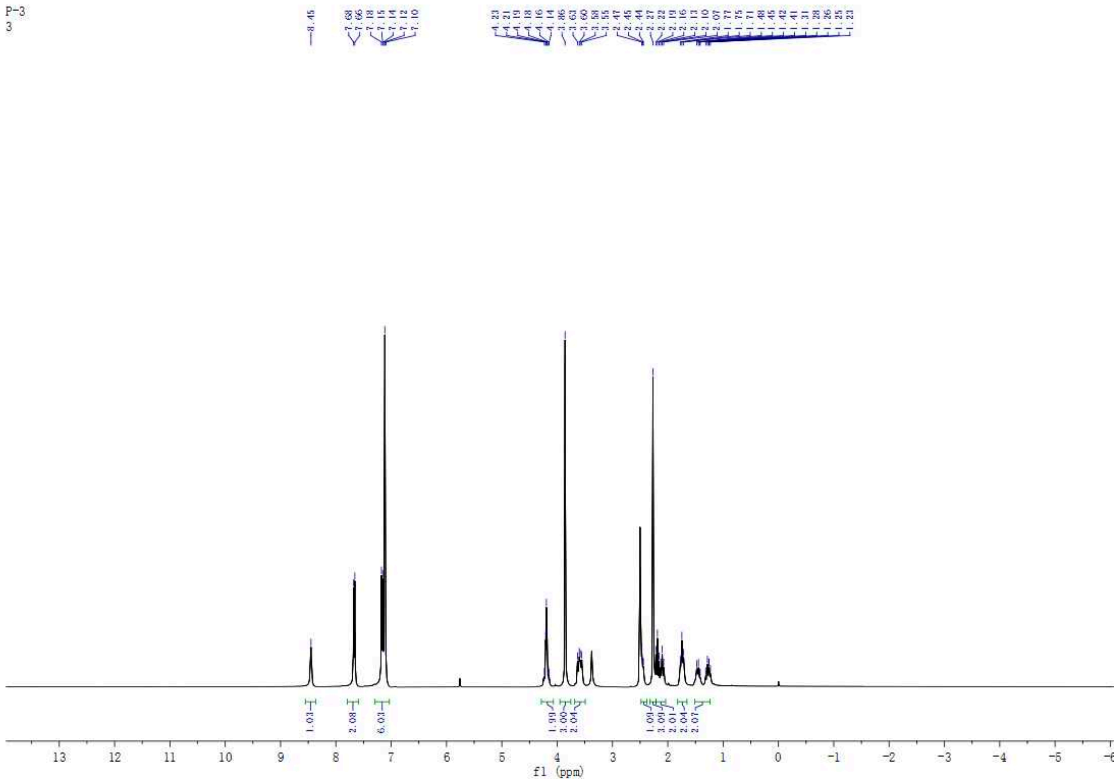

^{1}H NMR spectrum of compound H-3 (400 MHz, DMSO)

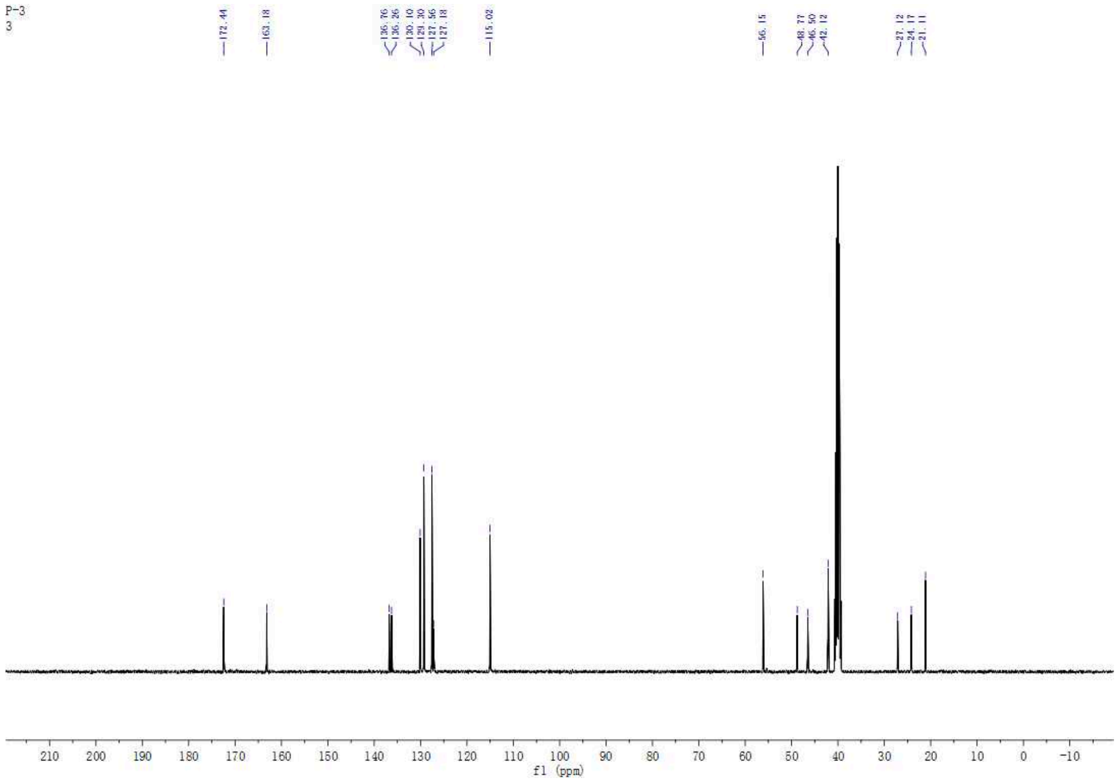

^{13}C NMR spectrum of compound H-3 (101 MHz, DMSO)

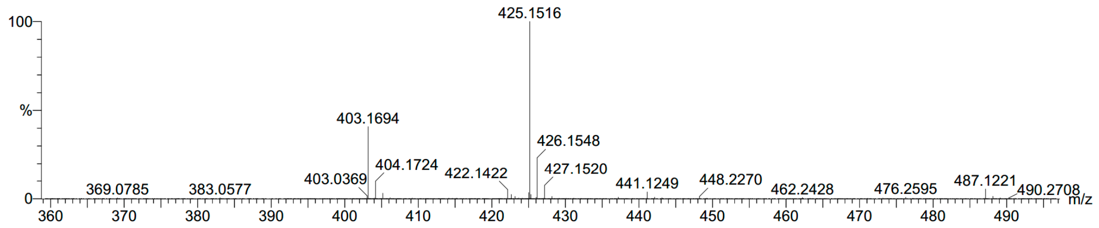

Minimum:				-1.5				
Maximum:		5.0	10.0	50.0				
Mass	Calc. Mass	mDa	PPM	DBE	i-FIT	Norm	Conf(%)	Formula
403.1694	403.1692	0.2	0.5	9.5	676.1	n/a	n/a	C21 H27 N2 O4 S

High resolution mass spectrum of compound H-3

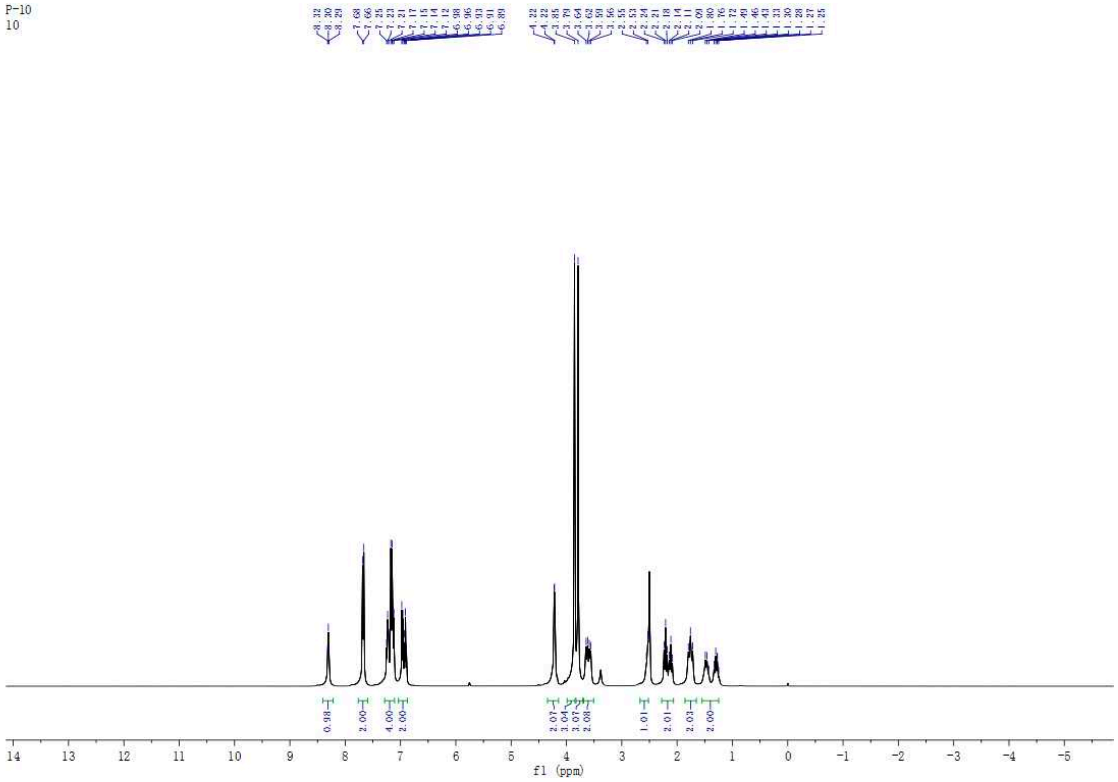

^{1}H NMR spectrum of compound H-4 (400 MHz, DMSO)

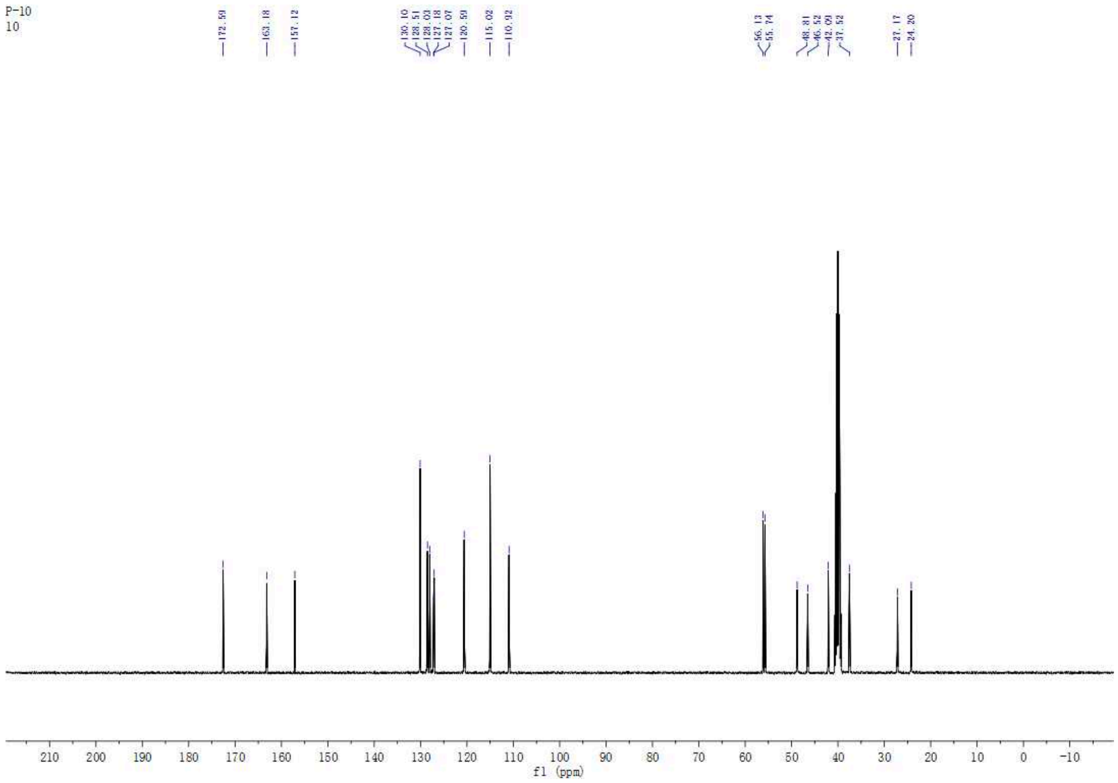

^{13}C NMR spectrum of compound H-4 (101 MHz, DMSO)

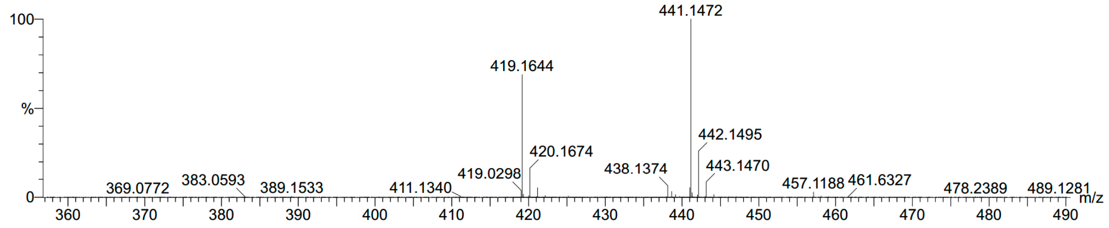

Minimum:				-1.5				
Maximum:		5.0	10.0	50.0				
Mass	Calc. Mass	mDa	PPM	DBE	i-FIT	Norm	Conf(%)	Formula
419.1644	419.1641	0.3	0.7	9.5	851.6	n/a	n/a	C21 H27 N2 O5 S

High resolution mass spectrum of compound H-4

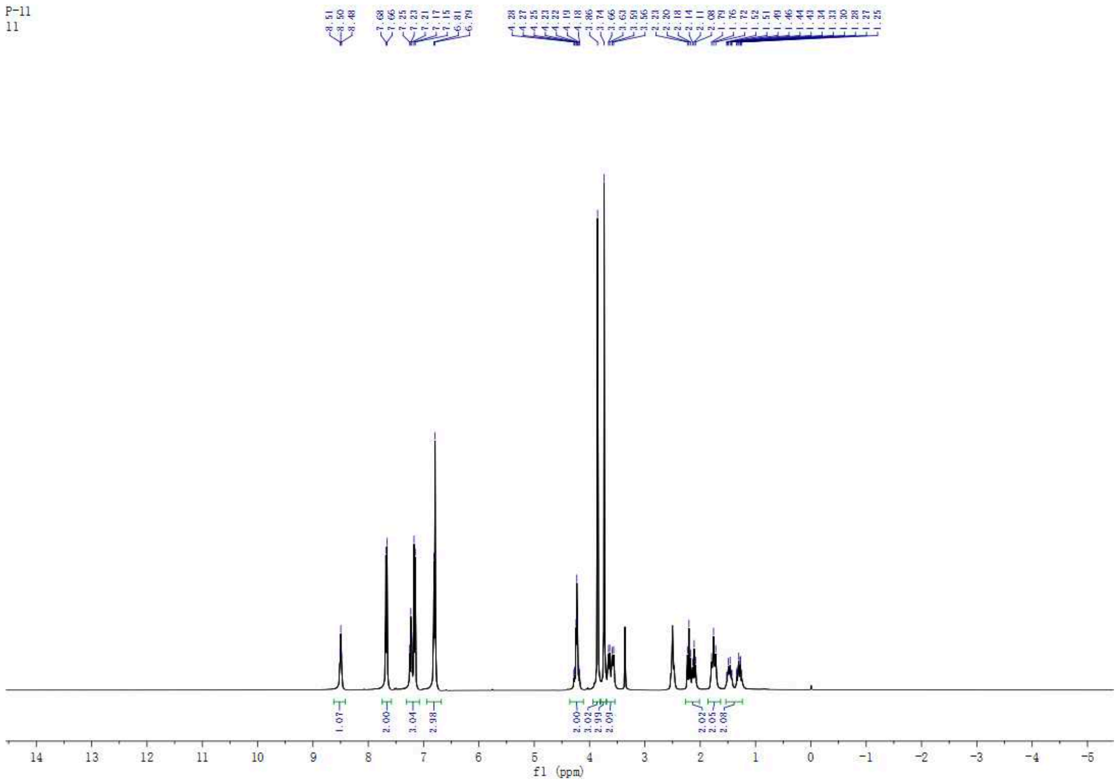

^{1}H NMR spectrum of compound H-5 (400 MHz, DMSO)

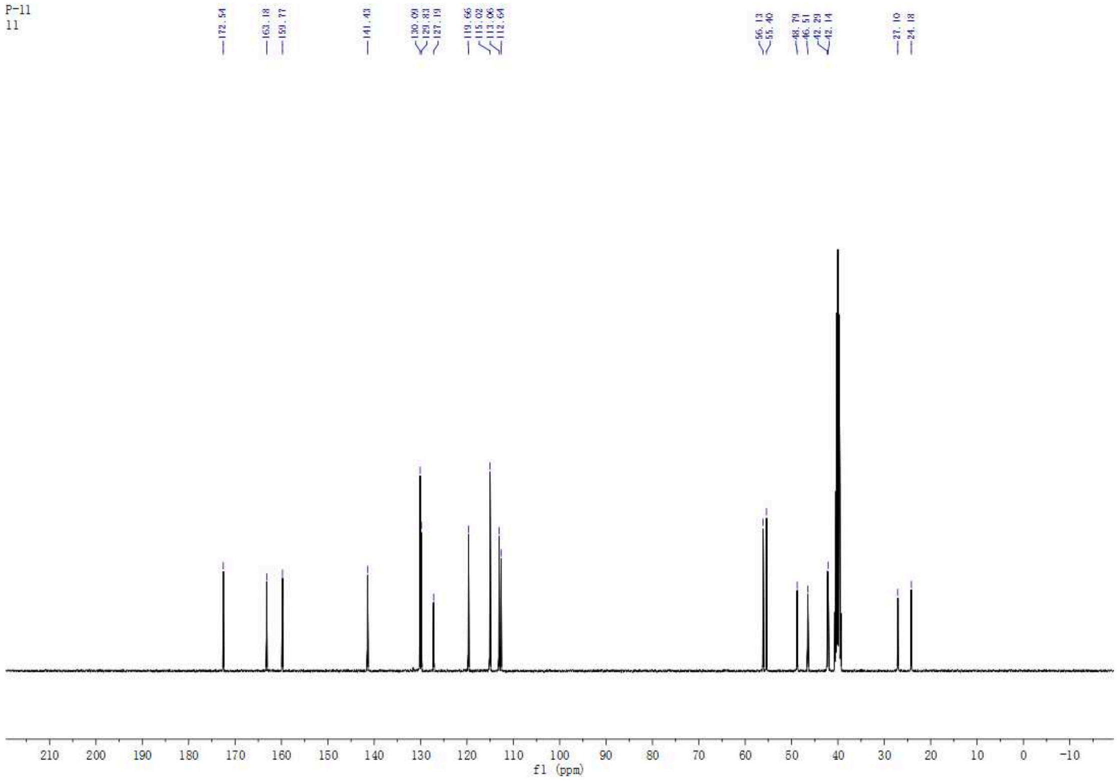

^{13}C NMR spectrum of compound H-5 (101 MHz, DMSO)

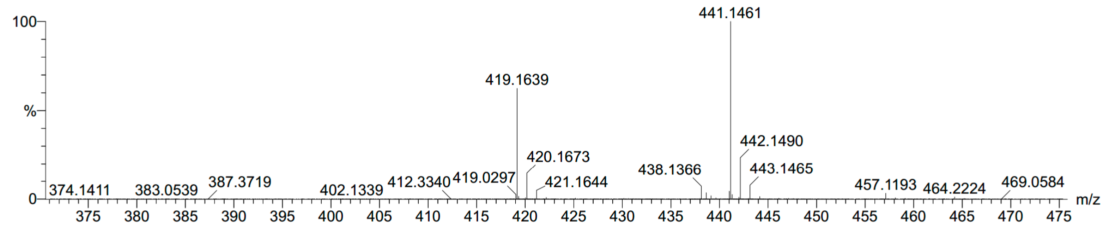

Minimum:				-1.5				
Maximum:		5.0	10.0	50.0				
Mass	Calc. Mass	mDa	PPM	DBE	i-FIT	Norm	Conf(%)	Formula
419.1639	419.1641	-0.2	-0.5	9.5	761.0	n/a	n/a	C21 H27 N2 O5 S

High resolution mass spectrum of compound H-5

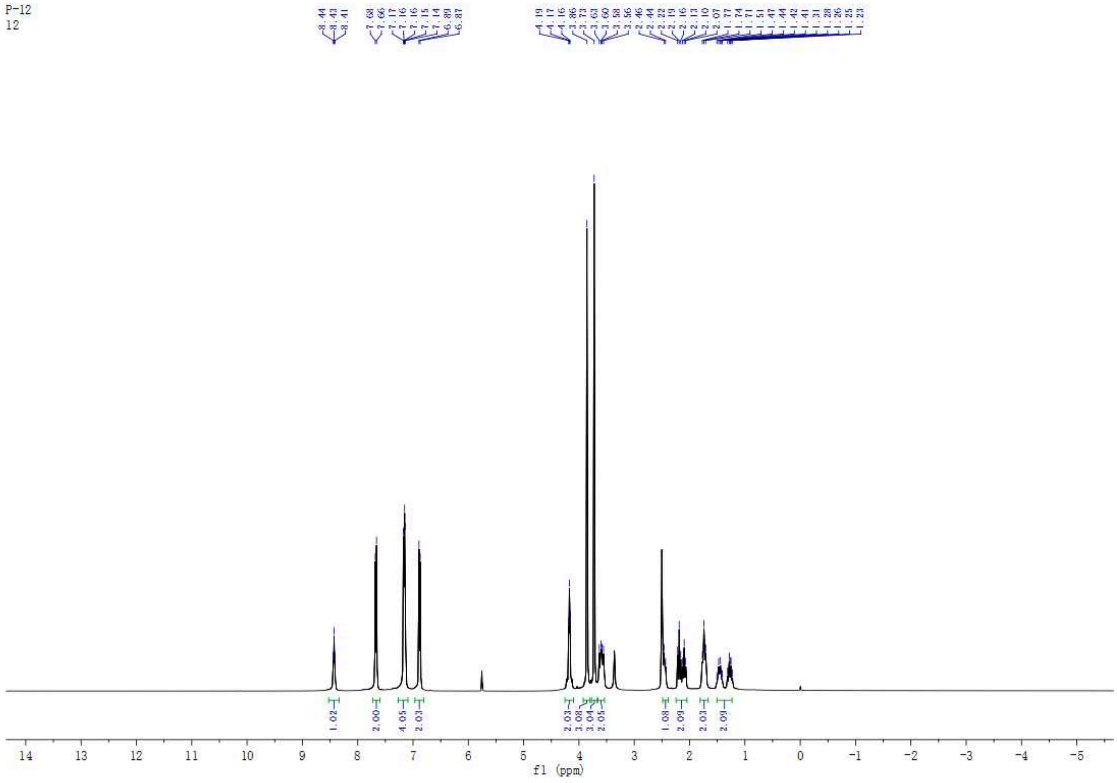

^{1}H NMR spectrum of compound H-6 (400 MHz, DMSO)

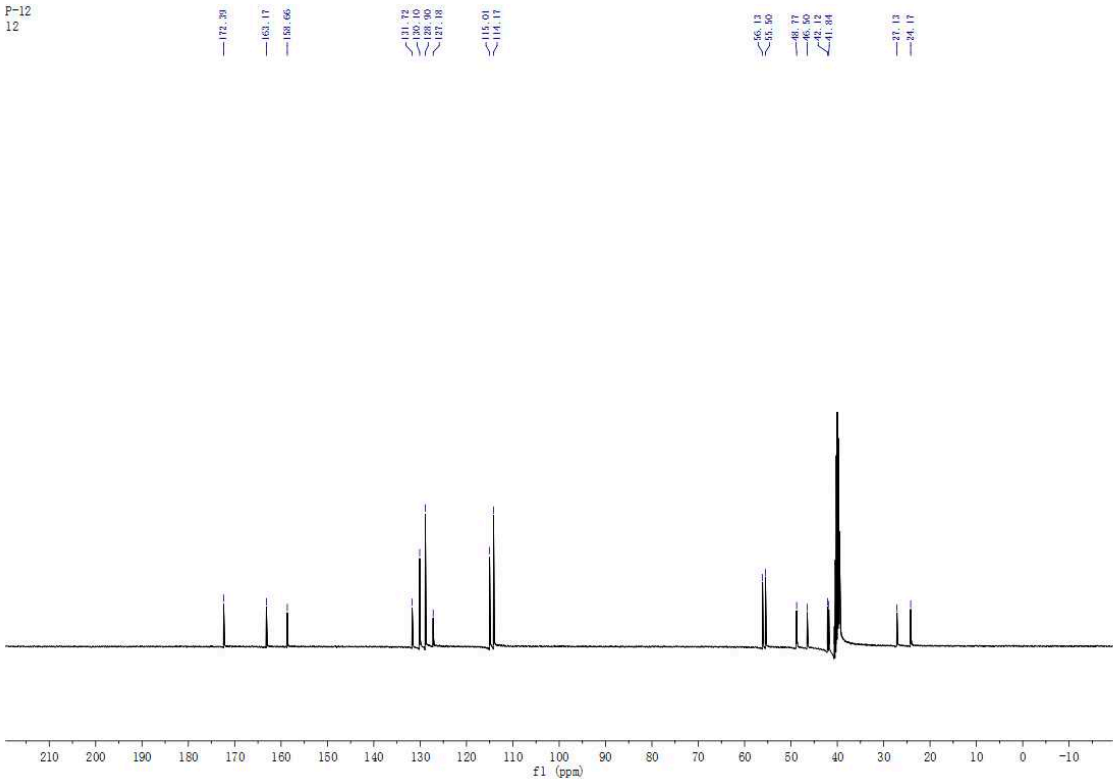

^{13}C NMR spectrum of compound H-6 (101 MHz, DMSO)

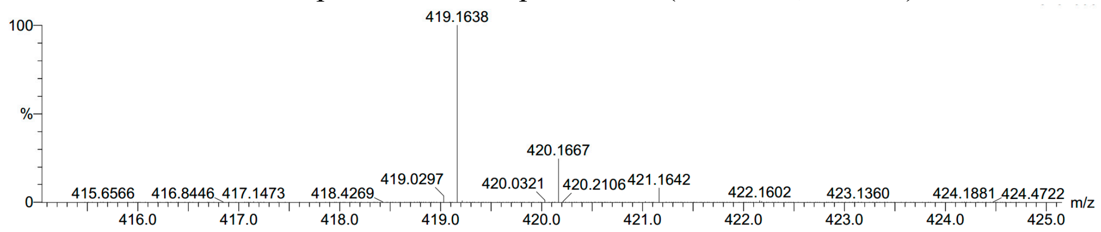

Minimum:				-1.5				
Maximum:		5.0	10.0	50.0				
Mass	Calc. Mass	mDa	PPM	DBE	i-FIT	Norm	Conf(%)	Formula
419.1638	419.1641	-0.3	-0.7	9.5	674.5	n/a	n/a	C21 H27 N2 O5 S

High resolution mass spectrum of compound H-6

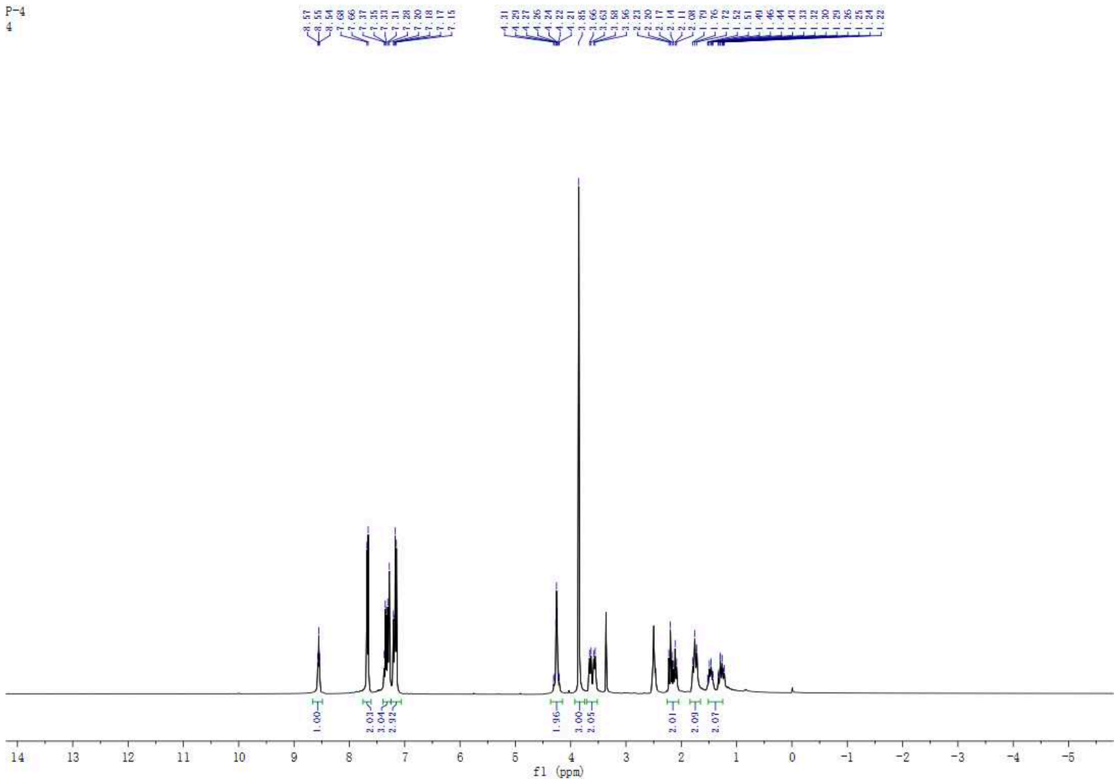

^{1}H NMR spectrum of compound H-7 (400 MHz, DMSO)

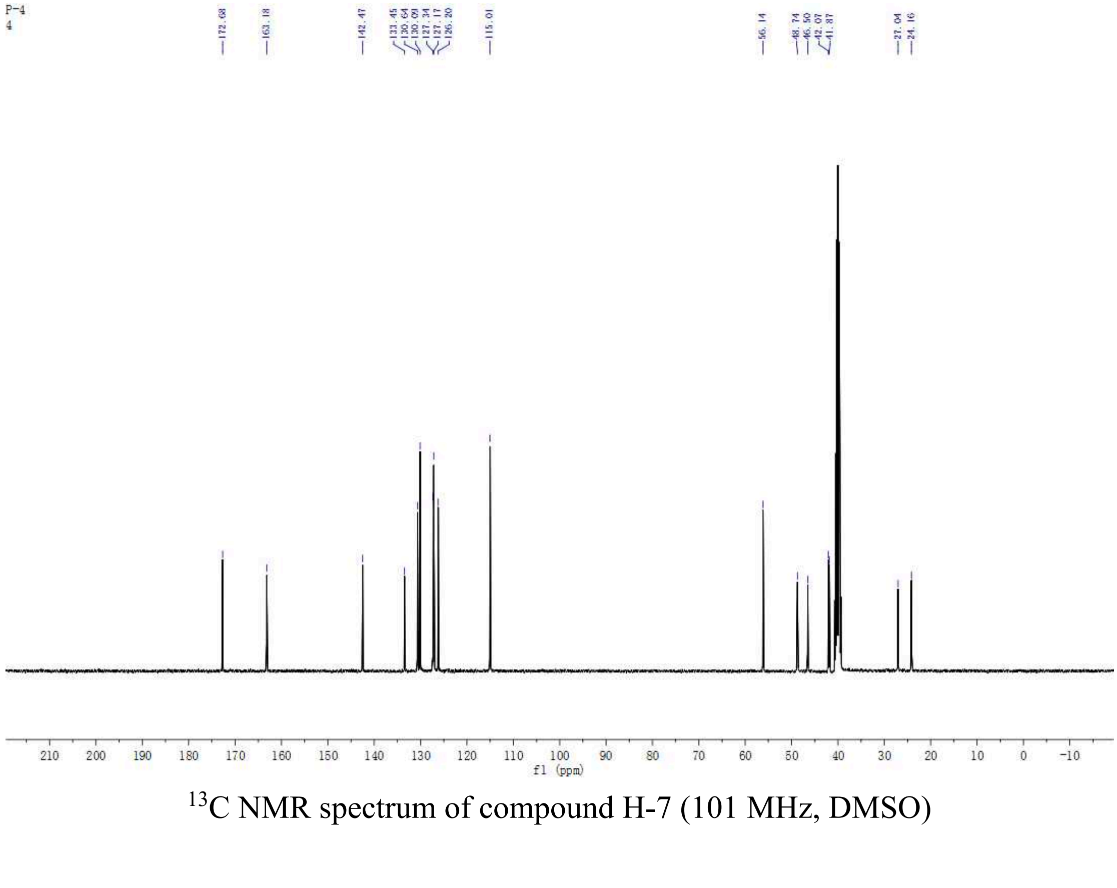

^{13}C NMR spectrum of compound H-7 (101 MHz, DMSO)

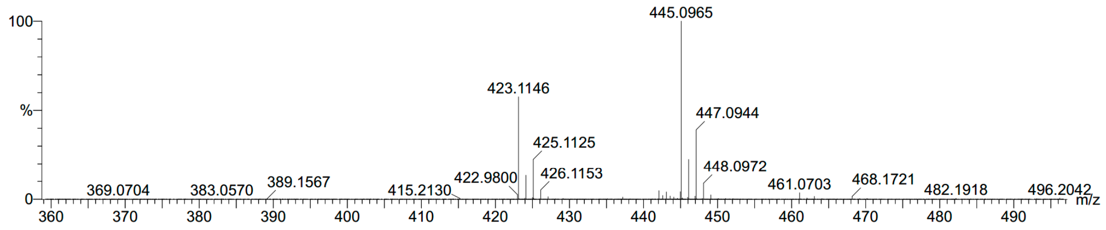

Minimum:				-1.5				
Maximum:		5.0	10.0	50.0				
Mass	Calc. Mass	mDa	PPM	DBE	i-FIT	Norm	Conf(%)	Formula
423.1146	423.1145	0.1	0.2	9.5	655.1	n/a	n/a	C20 H24 N2 O4 S Cl

High resolution mass spectrum of compound H-7

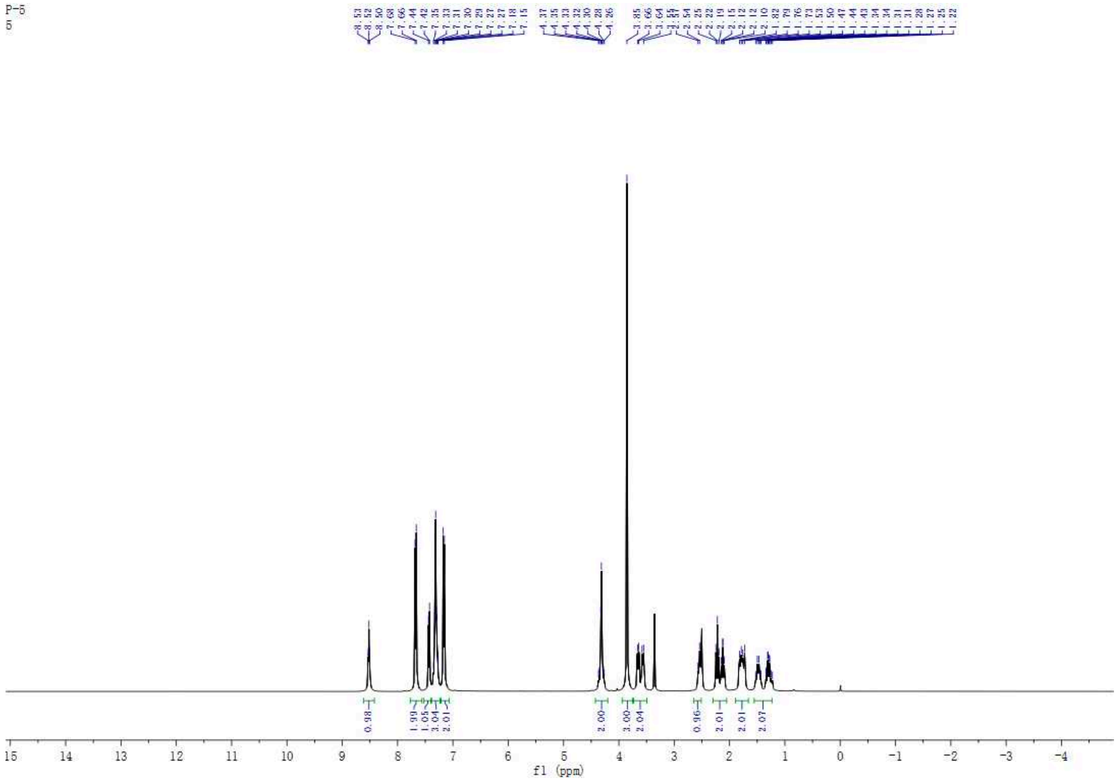

^{1}H NMR spectrum of compound H-8 (400 MHz, DMSO)

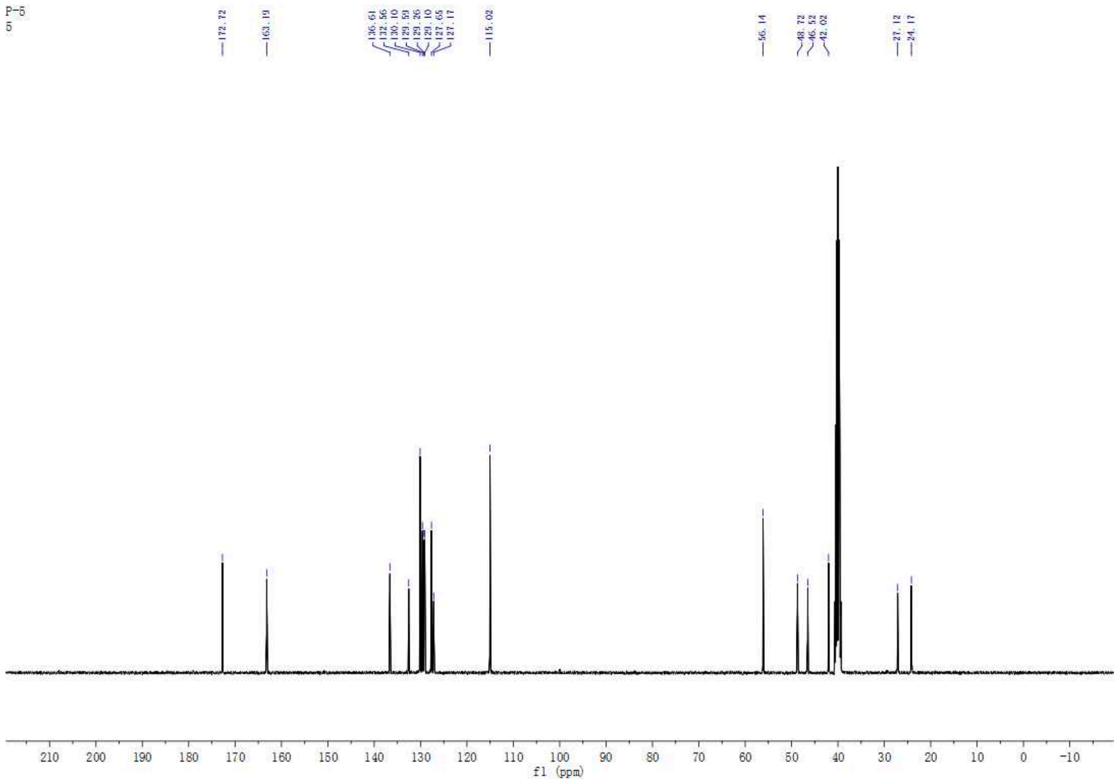

^{13}C NMR spectrum of compound H-8 (101 MHz, DMSO)

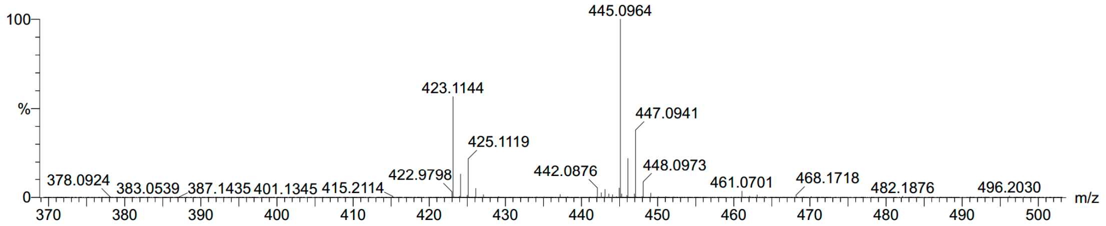

Minimum:				-1.5				
Maximum:		5.0	10.0	50.0				
Mass	Calc. Mass	mDa	PPM	DBE	i-FIT	Norm	Conf(%)	Formula
423.1144	423.1145	-0.1	-0.2	9.5	712.1	n/a	n/a	C20 H24 N2 O4 S Cl

High resolution mass spectrum of compound H-8

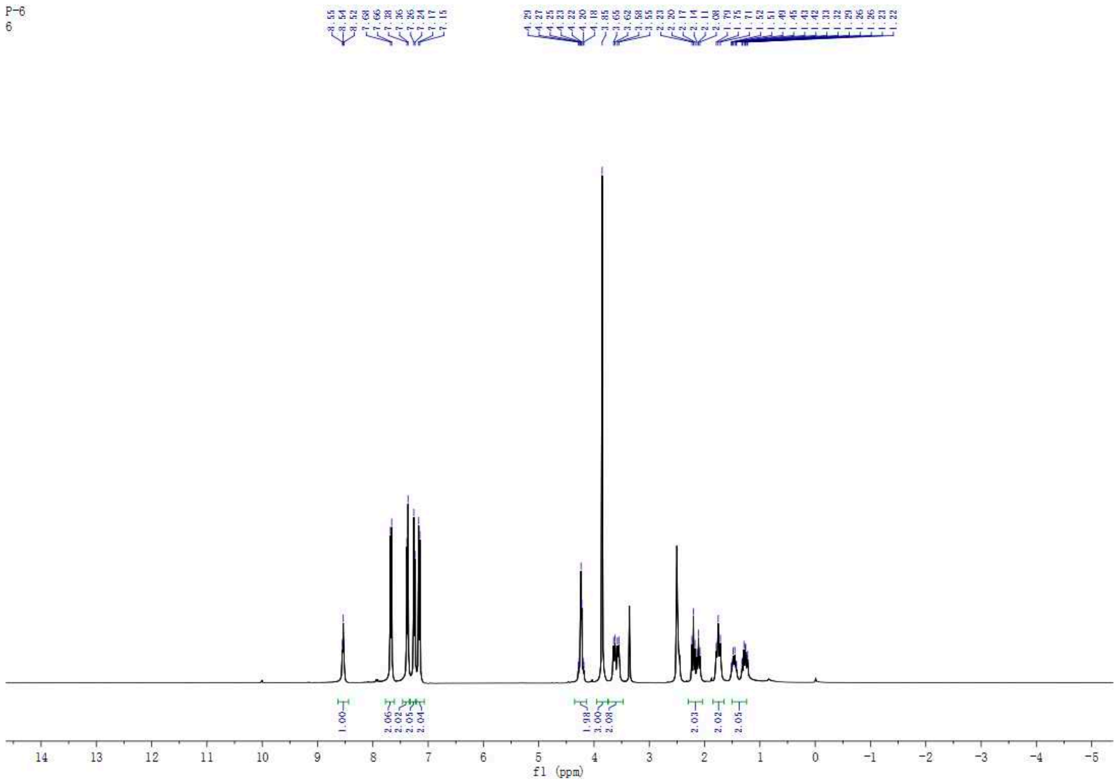

^{1}H NMR spectrum of compound H-9 (400 MHz, DMSO)

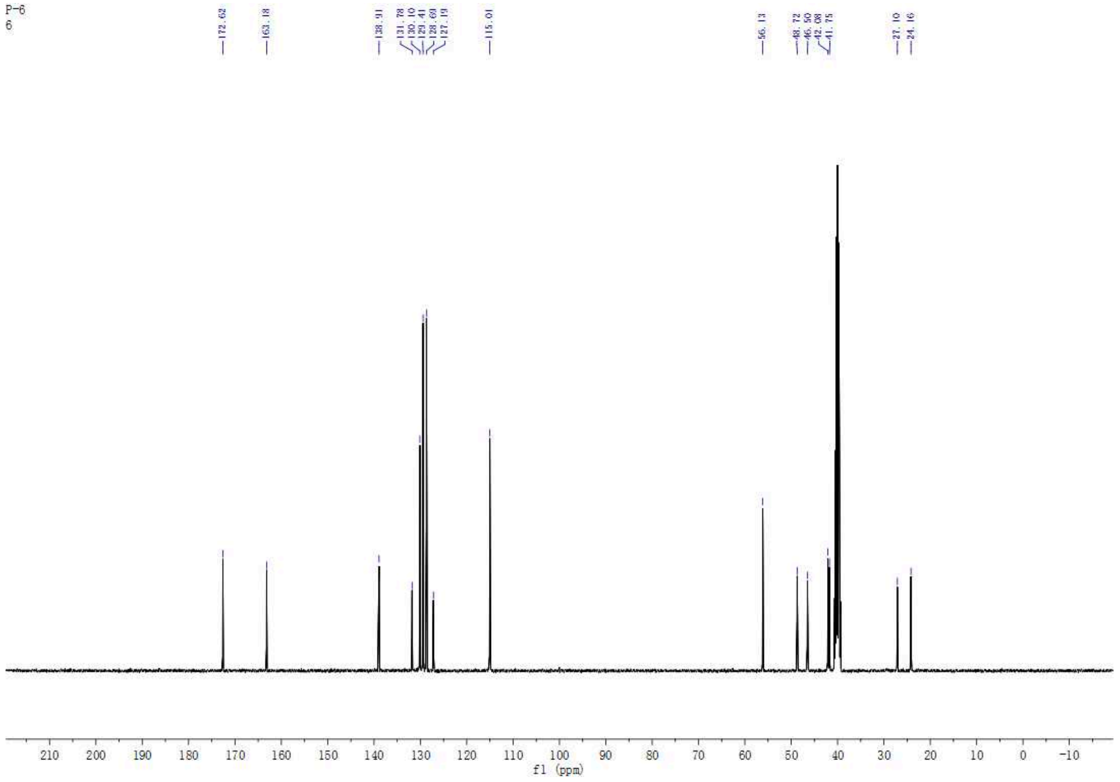

^{13}C NMR spectrum of compound H-9 (101 MHz, DMSO)

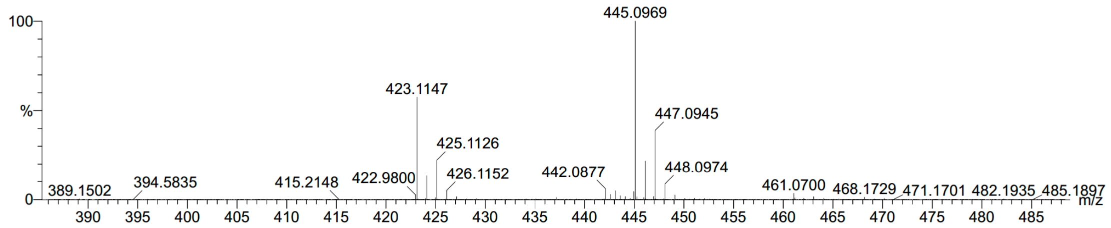

Minimum:				-1.5				
Maximum:		5.0	10.0	50.0				
Mass	Calc. Mass	mDa	PPM	DBE	i-FIT	Norm	Conf(%)	Formula
423.1147	423.1145	0.2	0.5	9.5	681.6	n/a	n/a	C20 H24 N2 O4 S Cl

High resolution mass spectrum of compound H-9

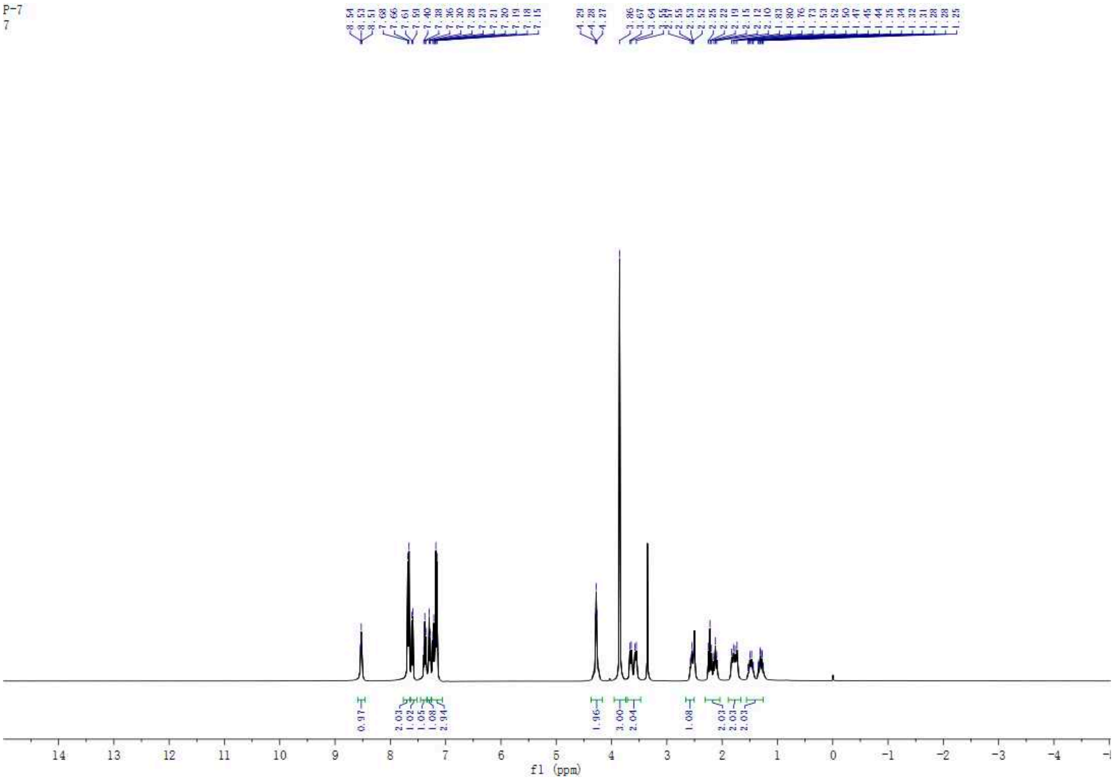

^{1}H NMR spectrum of compound H-10 (400 MHz, DMSO)

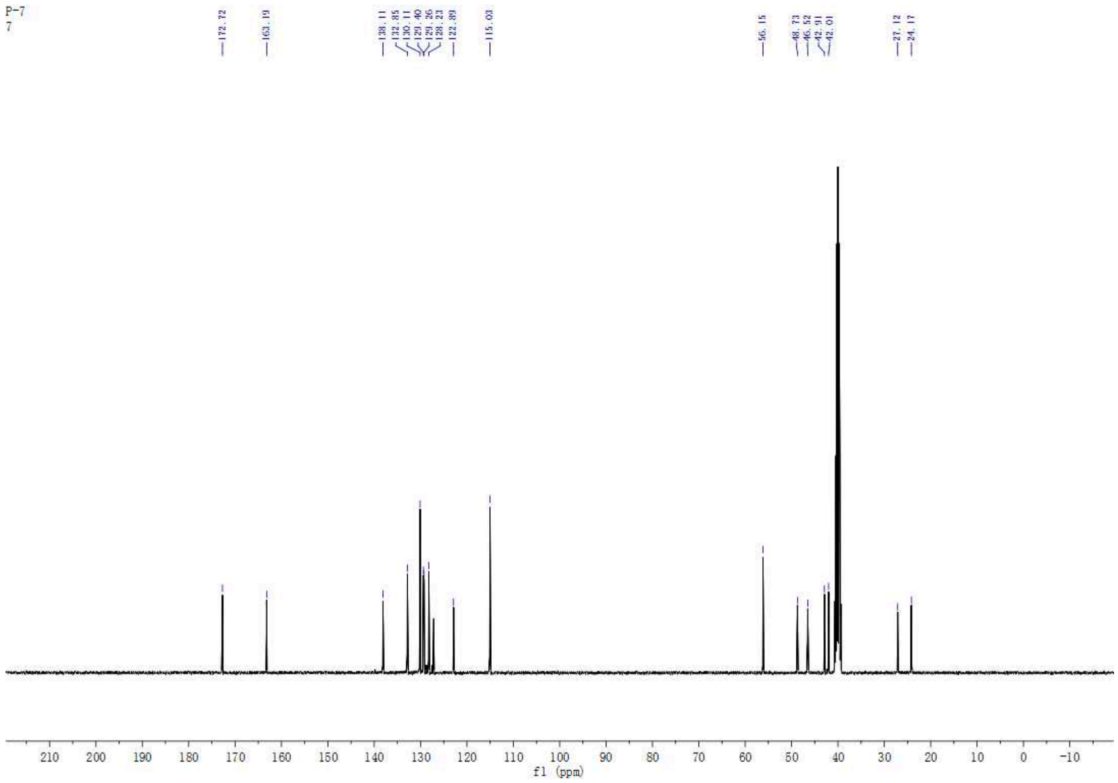

^{13}C NMR spectrum of compound H-10 (101 MHz, DMSO)

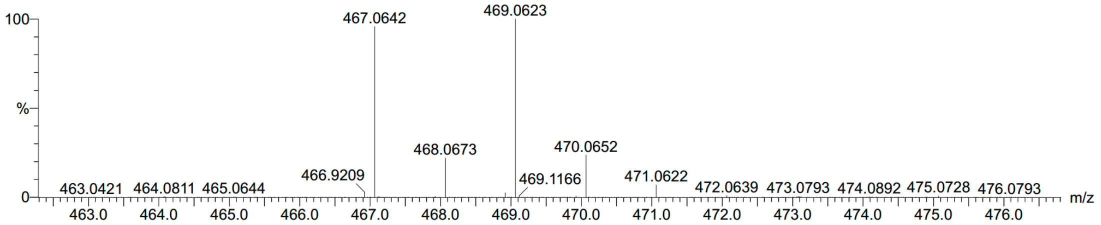

Minimum:				-1.5				
Maximum:		5.0	10.0	50.0				
Mass	Calc. Mass	mDa	PPM	DBE	i-FIT	Norm	Conf(%)	Formula
467.0642	467.0640	0.2	0.4	9.5	629.6	n/a	n/a	C20 H24 N2 O4 S Br

High resolution mass spectrum of compound H-10

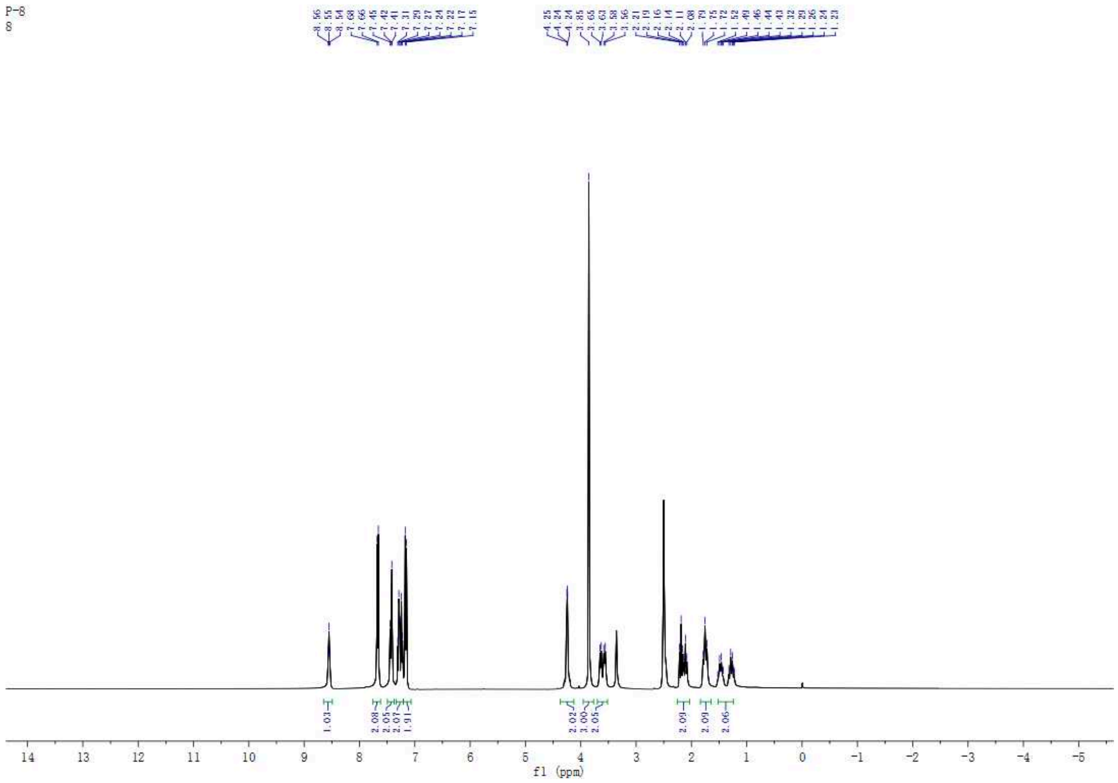

^{1}H NMR spectrum of compound H-11 (400 MHz, DMSO)

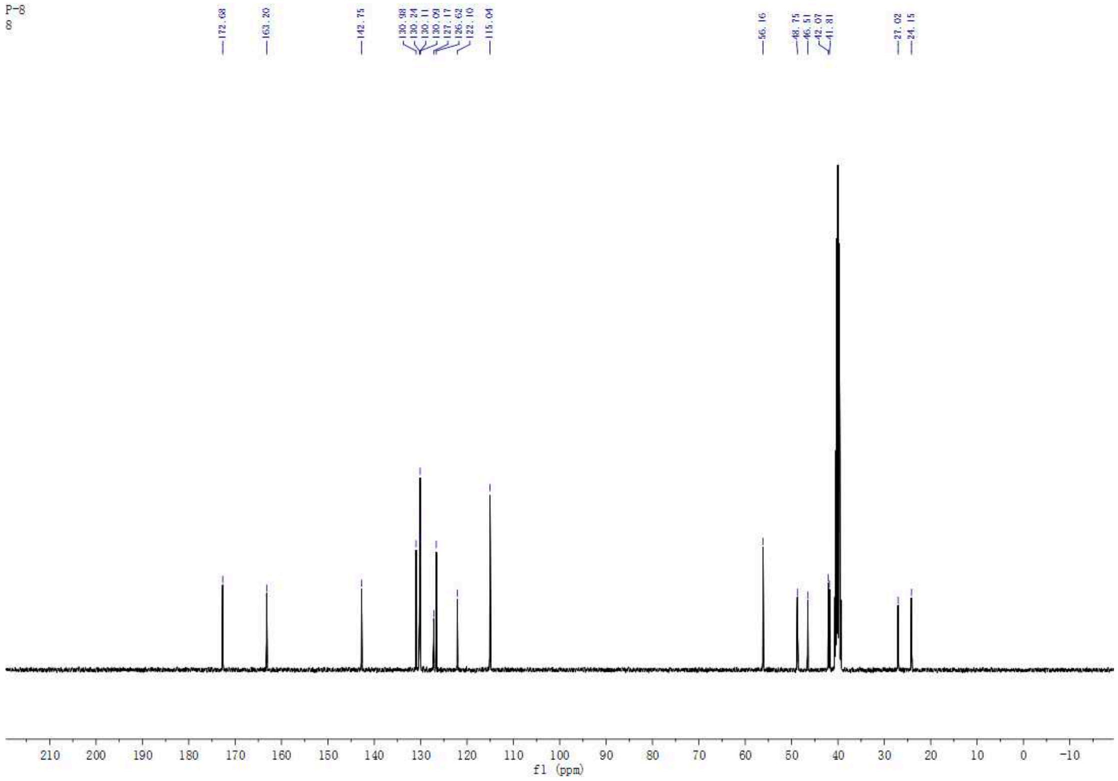

^{13}C NMR spectrum of compound H-11 (101 MHz, DMSO)

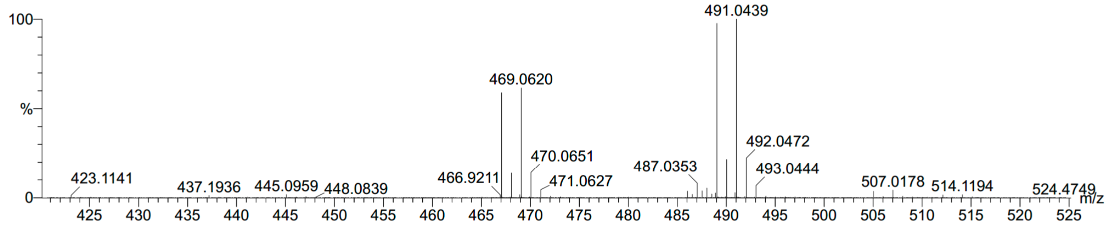

Minimum:				-1.5				
Maximum:		5.0	10.0	50.0				
Mass	Calc. Mass	mDa	PPM	DBE	i-FIT	Norm	Conf(%)	Formula
467.0639	467.0640	-0.1	-0.2	9.5	640.7	n/a	n/a	C20 H24 N2 O4 S Br

High resolution mass spectrum of compound H-11

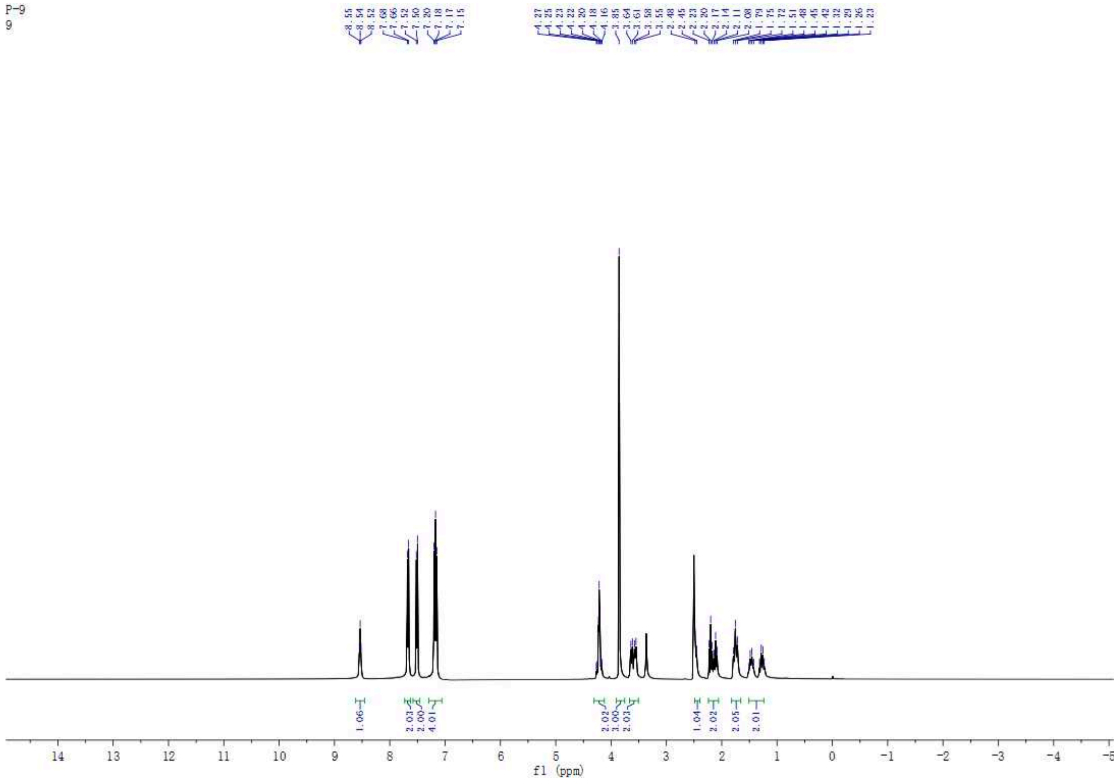

^{1}H NMR spectrum of compound H-12 (400 MHz, DMSO)

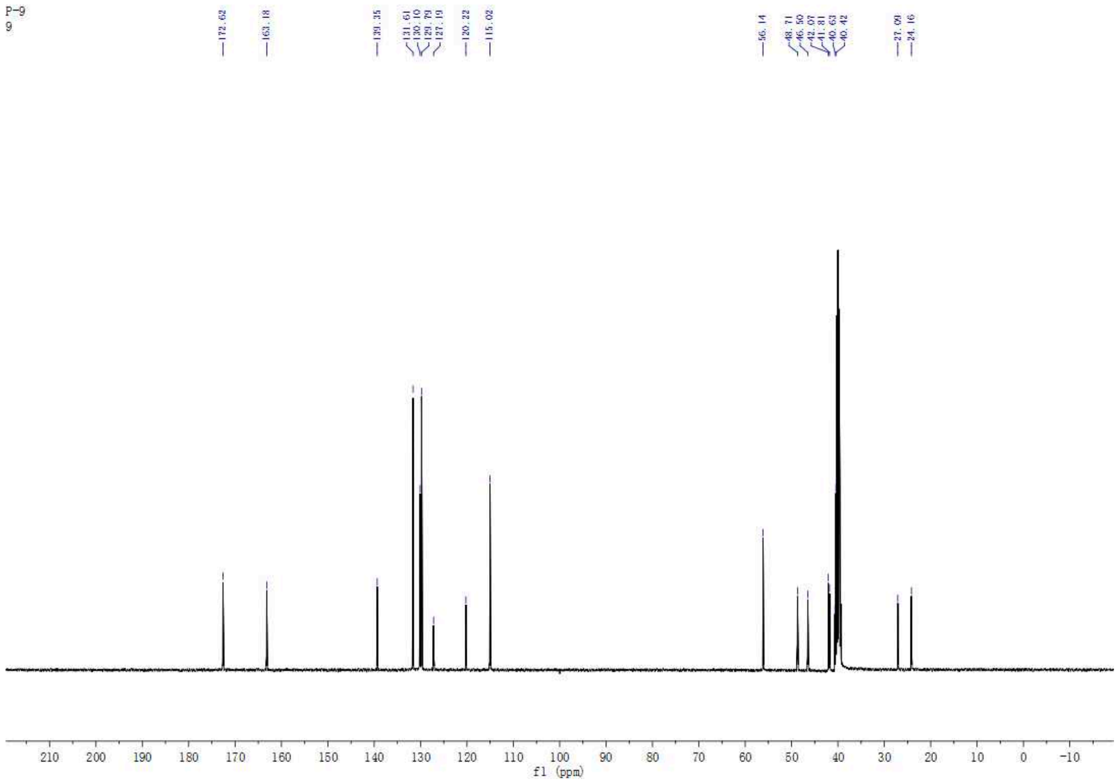

^{13}C NMR spectrum of compound H-12 (101 MHz, DMSO)

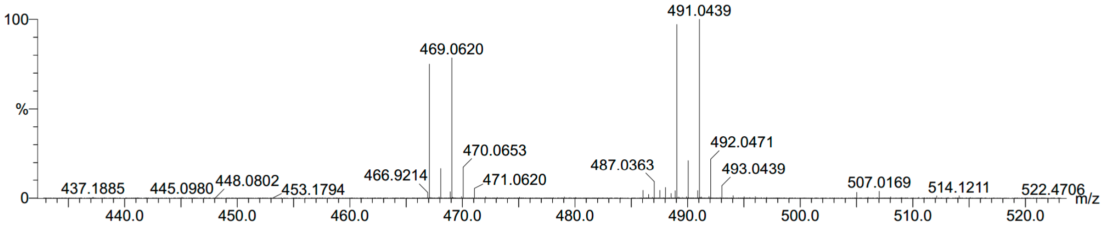

Minimum:				-1.5				
Maximum:		5.0	10.0	50.0				
Mass	Calc. Mass	mDa	PPM	DBE	i-FIT	Norm	Conf(%)	Formula
467.0638	467.0640	-0.2	-0.4	9.5	684.4	n/a	n/a	C20 H24 N2 O4 S Br

High resolution mass spectrum of compound H-12

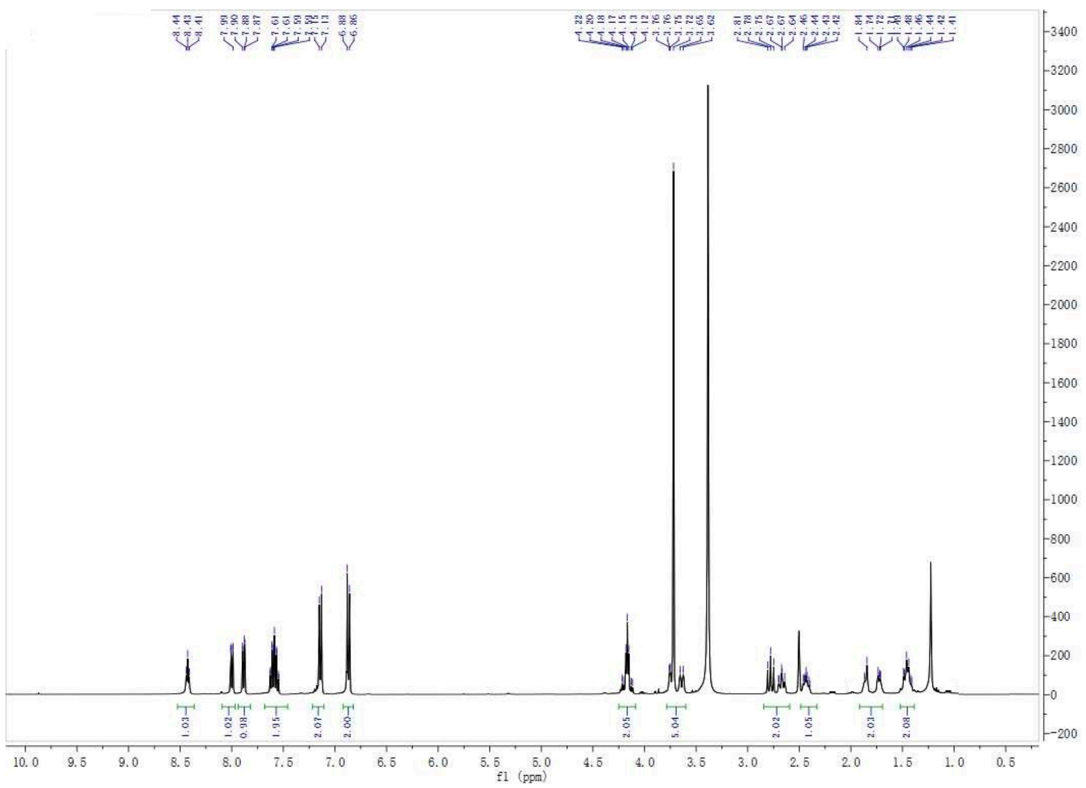

^{1}H NMR spectrum of compound H-13 (400 MHz, DMSO)

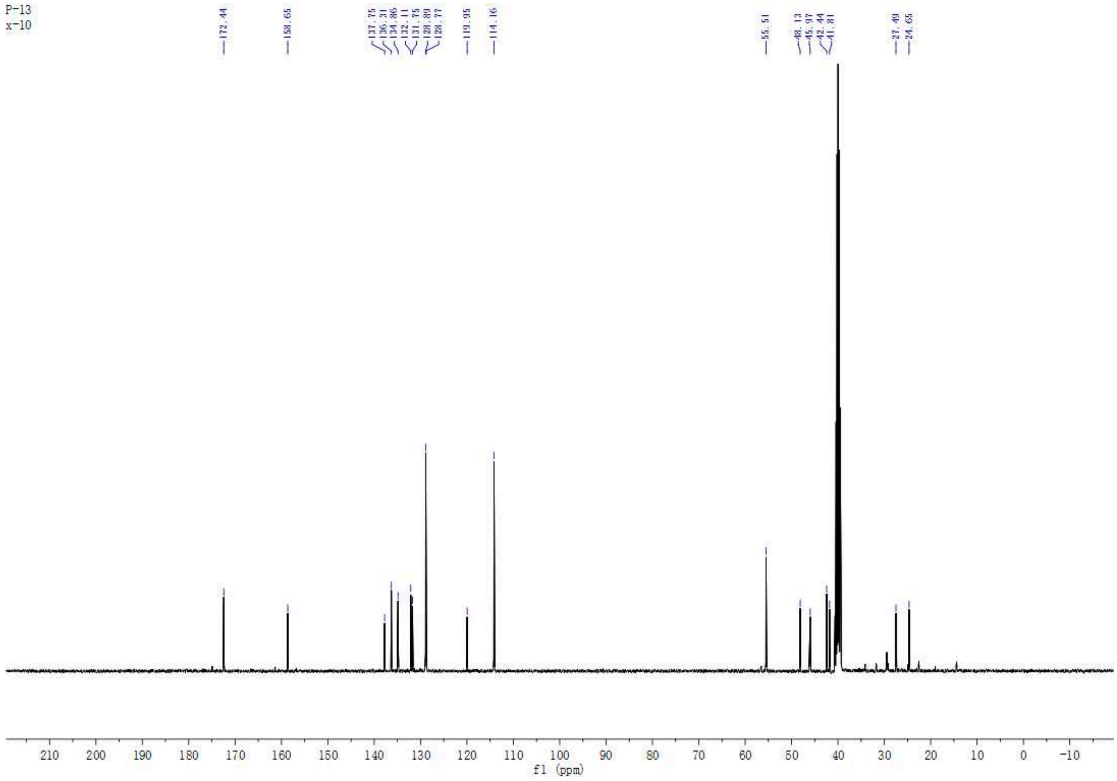

^{13}C NMR spectrum of compound H-13 (101 MHz, DMSO)

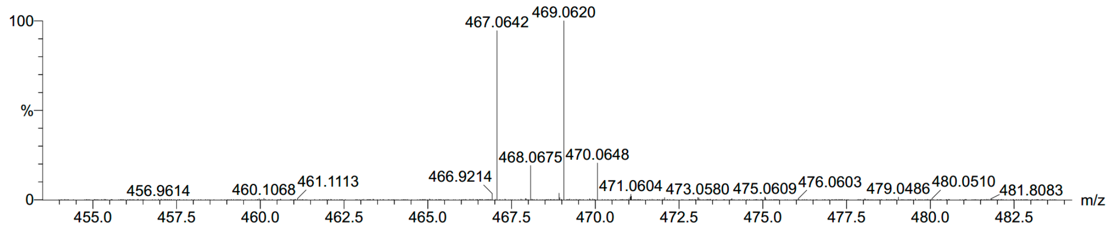

	Calc. Mass	mDa	PPM	DBE	i-FIT	Norm	Conf(%)	Formula
Minimum:				-1.5				
Maximum:		5.0	10.0	50.0				

Mass	Calc. Mass	mDa	PPM	DBE	i-FIT	Norm	Conf(%)	Formula
467.0642	467.0640	0.2	0.4	9.5	400.2	n/a	n/a	C20 H24 N2 O4 S Br

High resolution mass spectrum of compound H-13

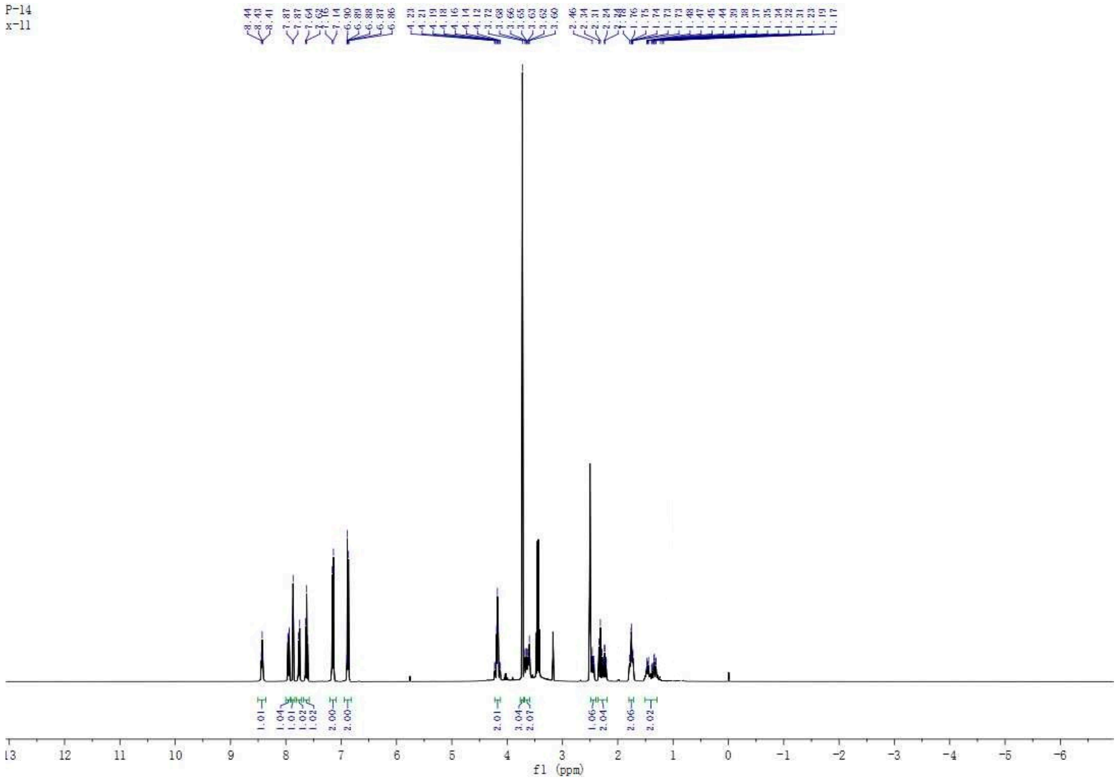

^{1}H NMR spectrum of compound H-14 (400 MHz, DMSO)

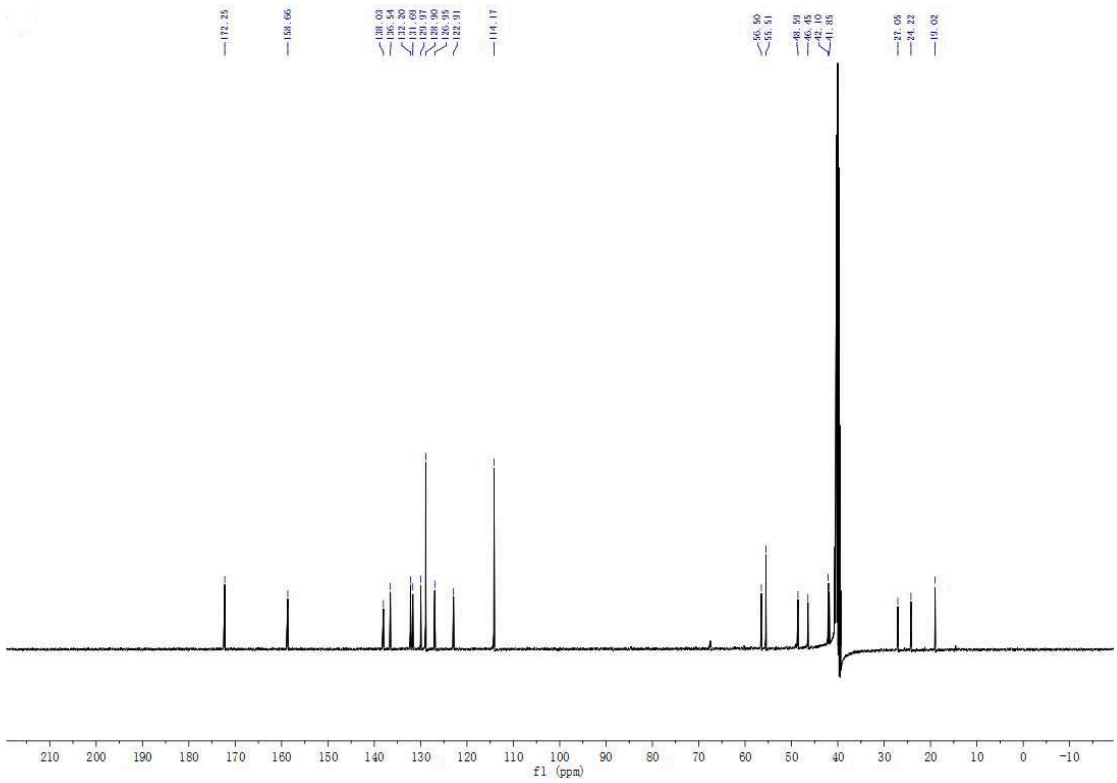

^{13}C NMR spectrum of compound H-14 (101 MHz, DMSO)

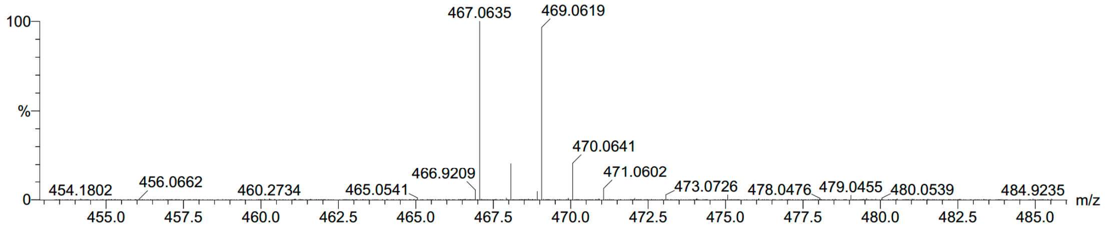

Minimum:				-1.5				
Maximum:		5.0	10.0	50.0				
Mass	Calc. Mass	mDa	PPM	DBE	i-FIT	Norm	Conf(%)	Formula
467.0635	467.0640	-0.5	-1.1	9.5	510.3	n/a	n/a	C20 H24 N2 O4 S Br

High resolution mass spectrum of compound H-14

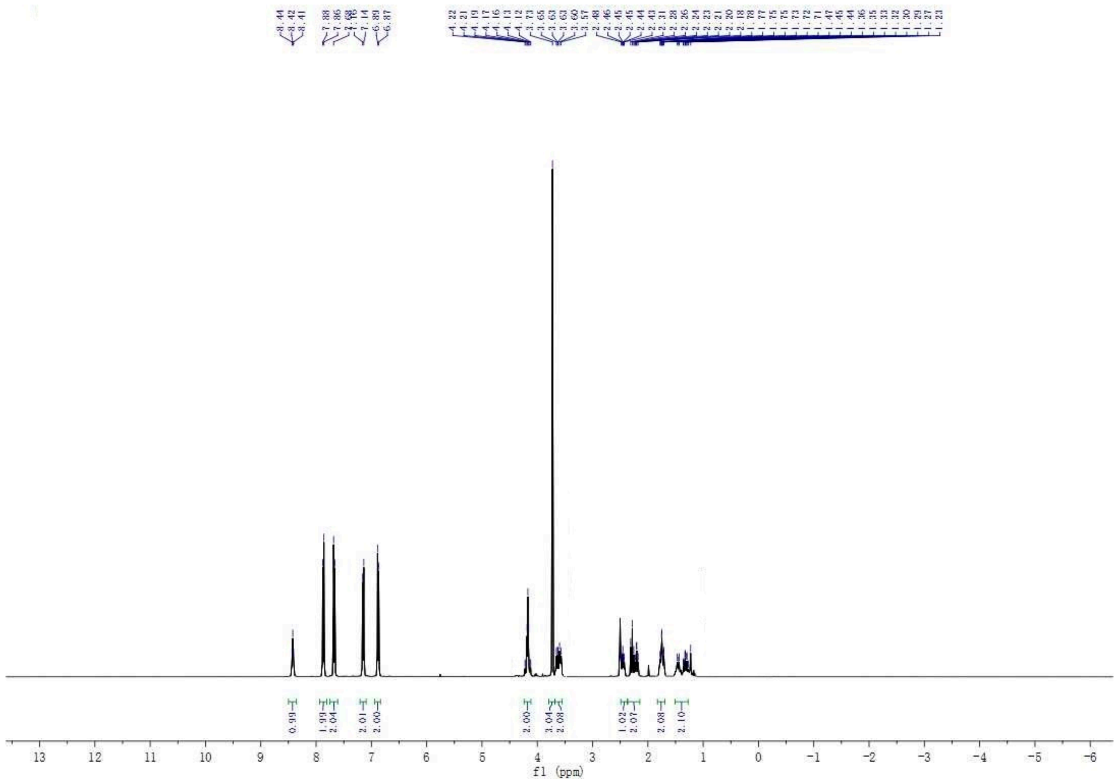

^{1}H NMR spectrum of compound H-15 (400 MHz, DMSO)

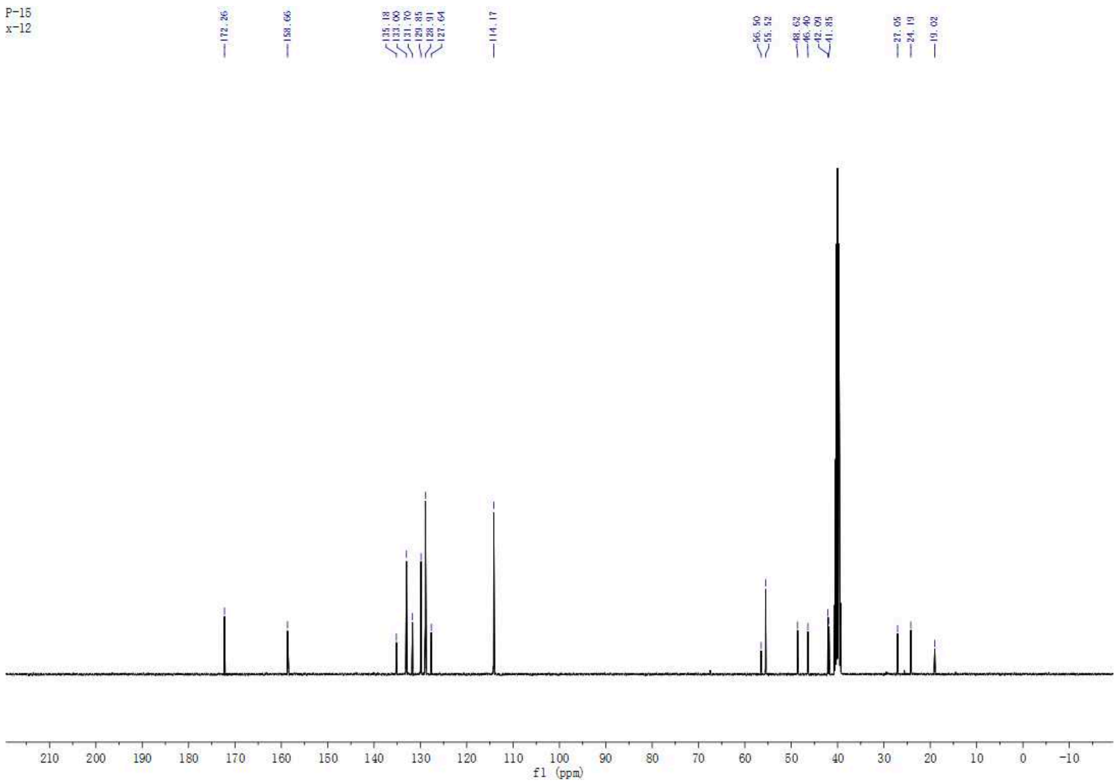

^{13}C NMR spectrum of compound H-15 (101 MHz, DMSO)

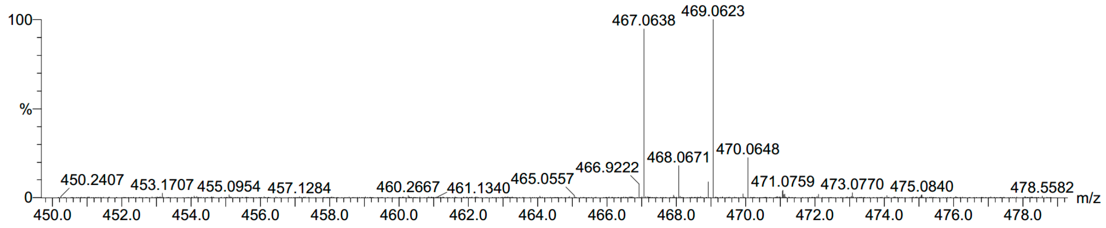

	Mass	Calc. Mass	mDa	PPM	DBE	i-FIT	Norm	Conf(%)	Formula
Minimum:					-1.5				
Maximum:			5.0	10.0	50.0				
	467.0638	467.0640	-0.2	-0.4	9.5	495.7	n/a	n/a	C20 H24 N2 O4 S Br

High resolution mass spectrum of compound H-15

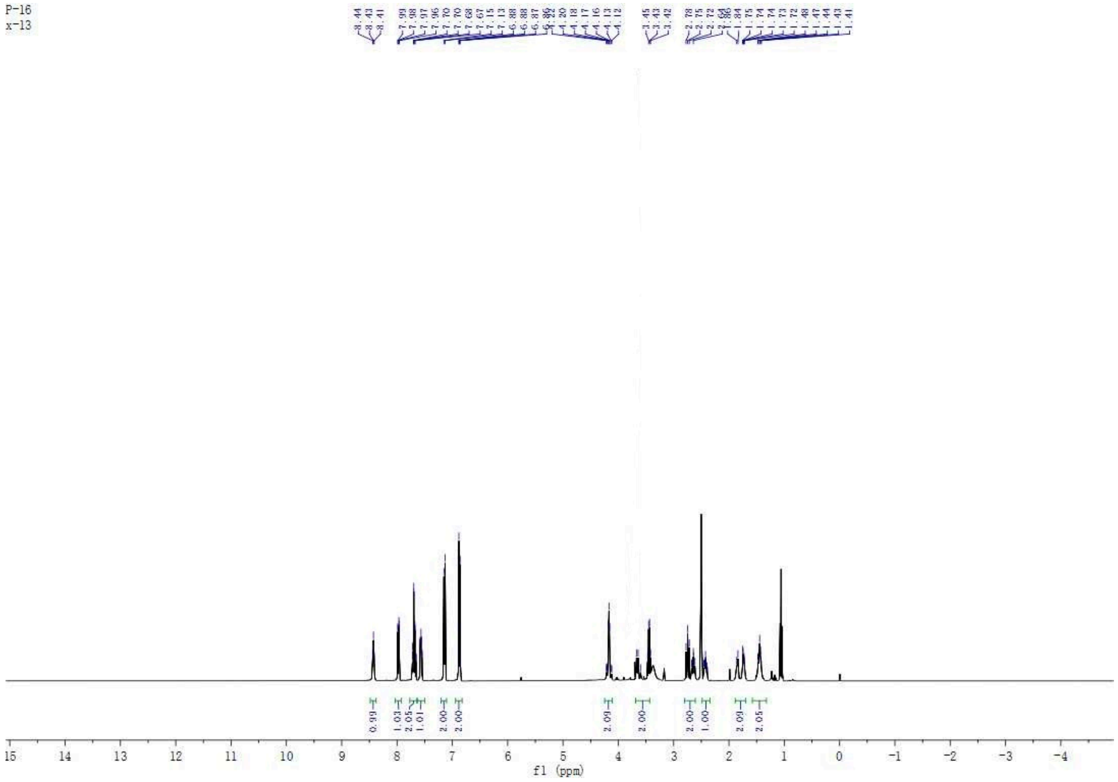

^{1}H NMR spectrum of compound H-16 (400 MHz, DMSO)

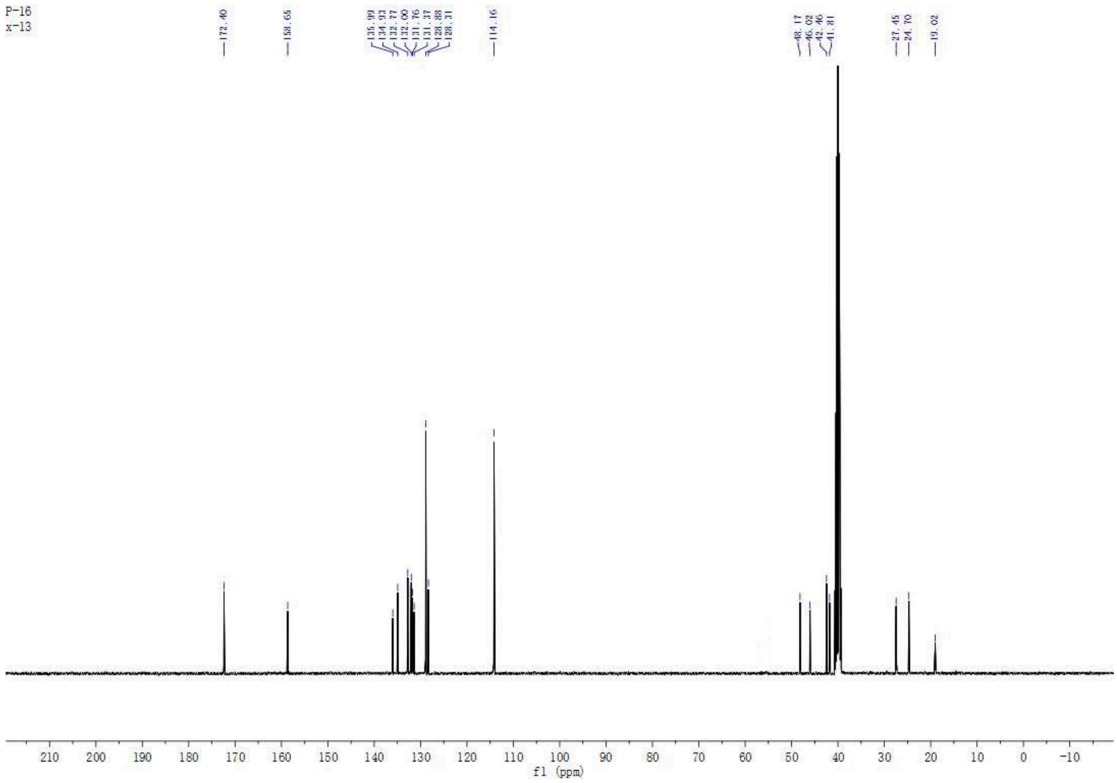

^{13}C NMR spectrum of compound H-16 (101 MHz, DMSO)

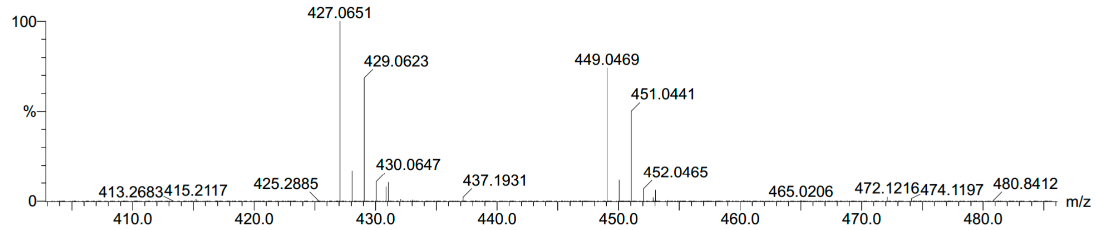

Minimum:				-1.5				
Maximum:		5.0	20.0	50.0				
Mass	Calc. Mass	mDa	PPM	DBE	i-FIT	Norm	Conf(%)	Formula
427.0651	427.0650	0.1	0.2	9.5	321.1	n/a	n/a	C19 H21 N2 O3 S Cl2

High resolution mass spectrum of compound H-16

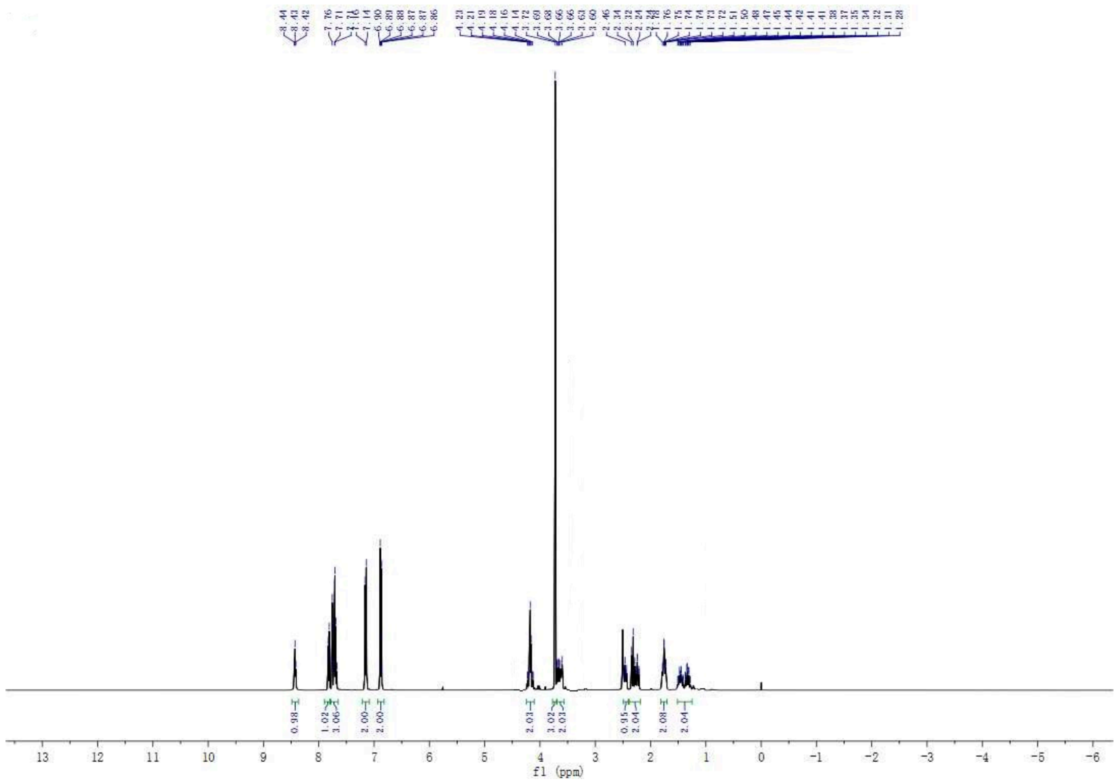

^{1}H NMR spectrum of compound H-17 (400 MHz, DMSO)

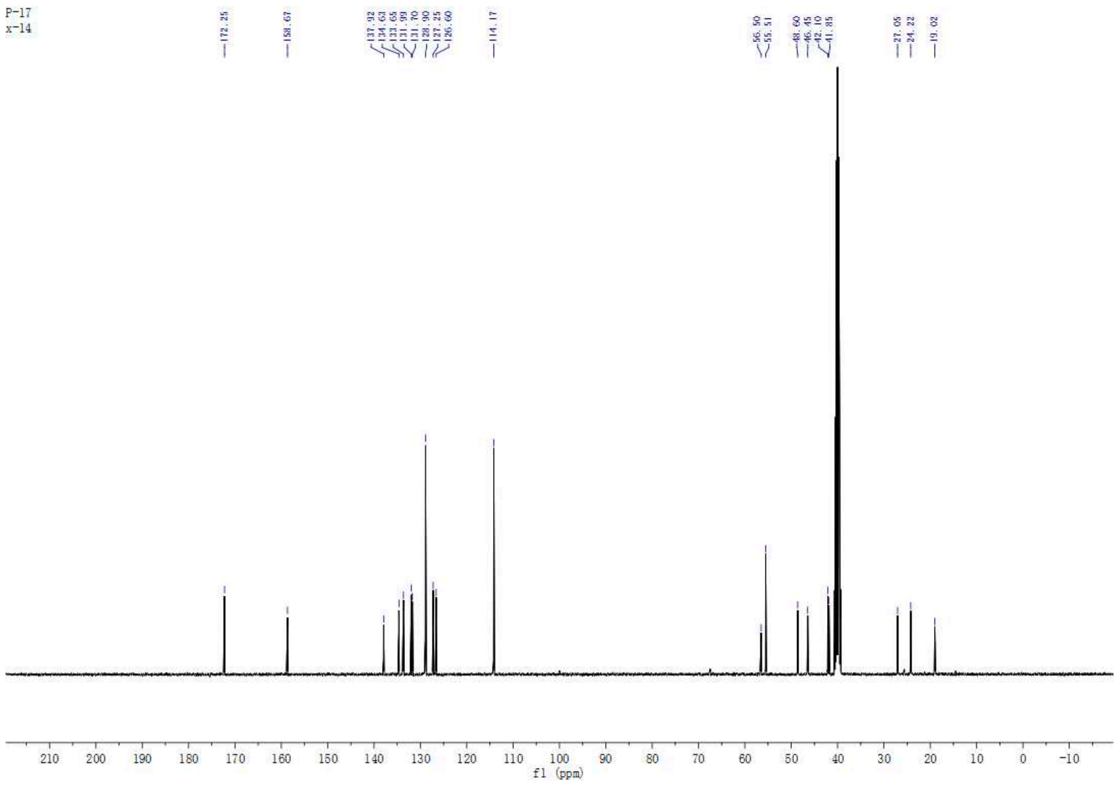

^{13}C NMR spectrum of compound H-17 (101 MHz, DMSO)

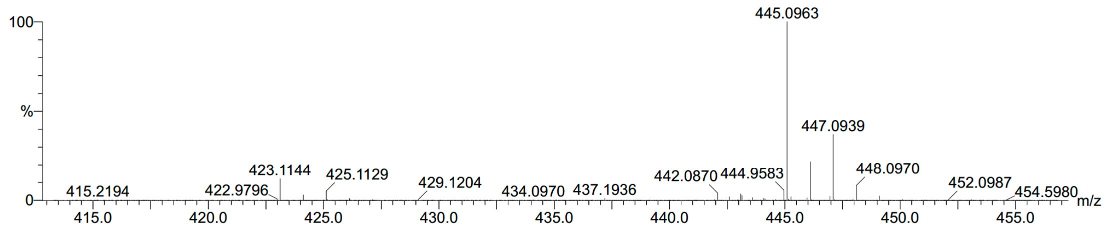

Minimum:				-1.5				
Maximum:		5.0	10.0	50.0				
Mass	Calc. Mass	mDa	PPM	DBE	i-FIT	Norm	Conf(%)	Formula
423.1144	423.1145	-0.1	-0.2	9.5	628.4	n/a	n/a	C20 H24 N2 O4 S Cl

High resolution mass spectrum of compound H-17

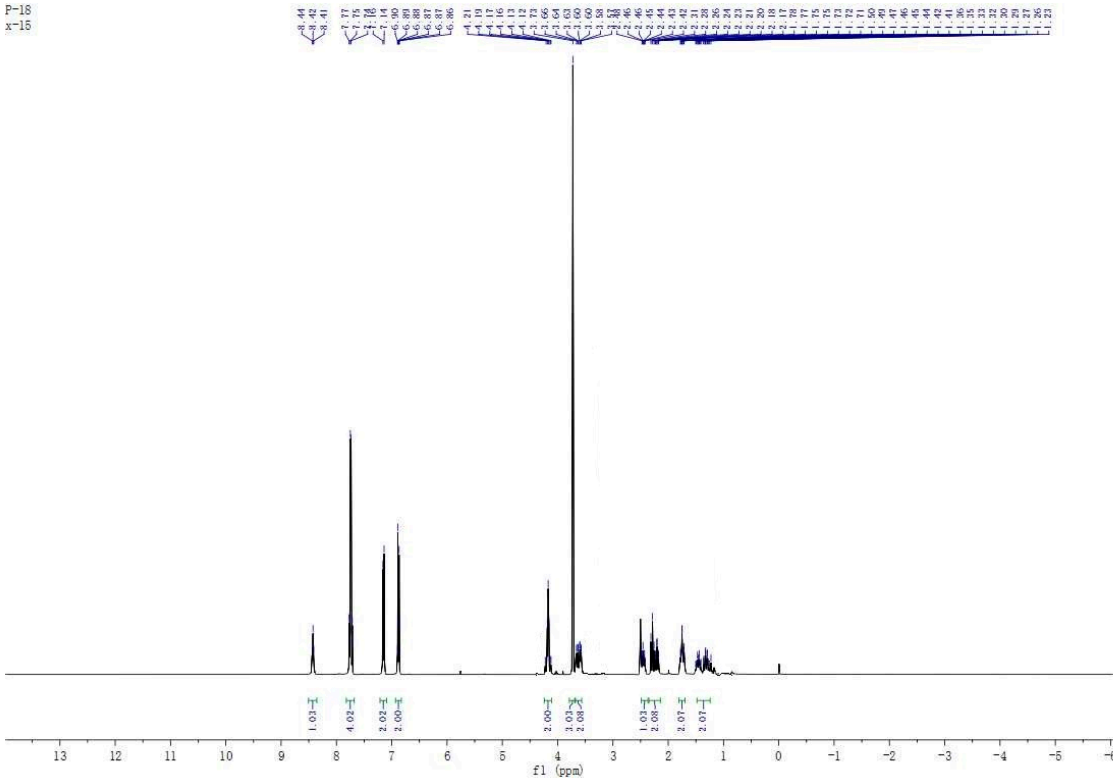

^{1}H NMR spectrum of compound H-18 (400 MHz, DMSO)

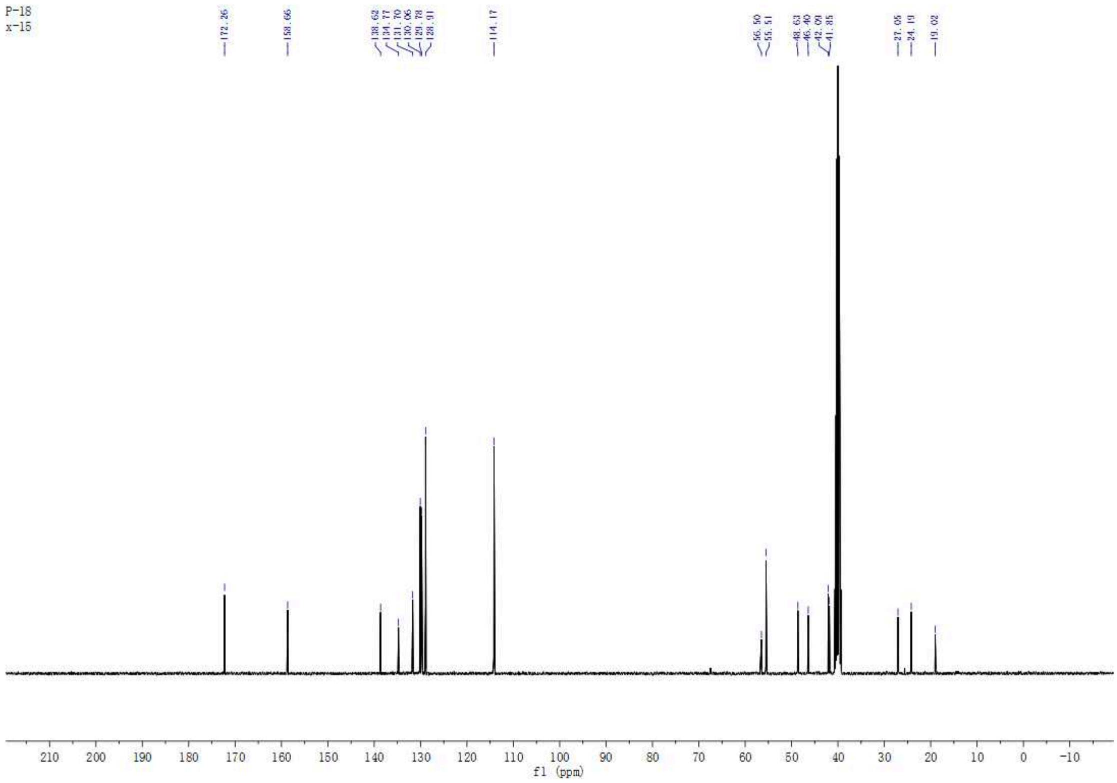

^{13}C NMR spectrum of compound H-18 (101 MHz, DMSO)

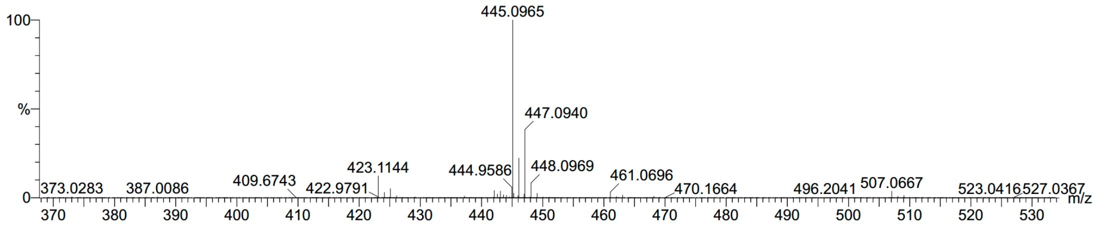

Minimum:				-1.5				
Maximum:		5.0	10.0	50.0				
Mass	Calc. Mass	mDa	PPM	DBE	i-FIT	Norm	Conf(%)	Formula
423.1144	423.1145	-0.1	-0.2	9.5	697.2	n/a	n/a	C20 H24 N2 O4 S Cl

High resolution mass spectrum of compound H-18

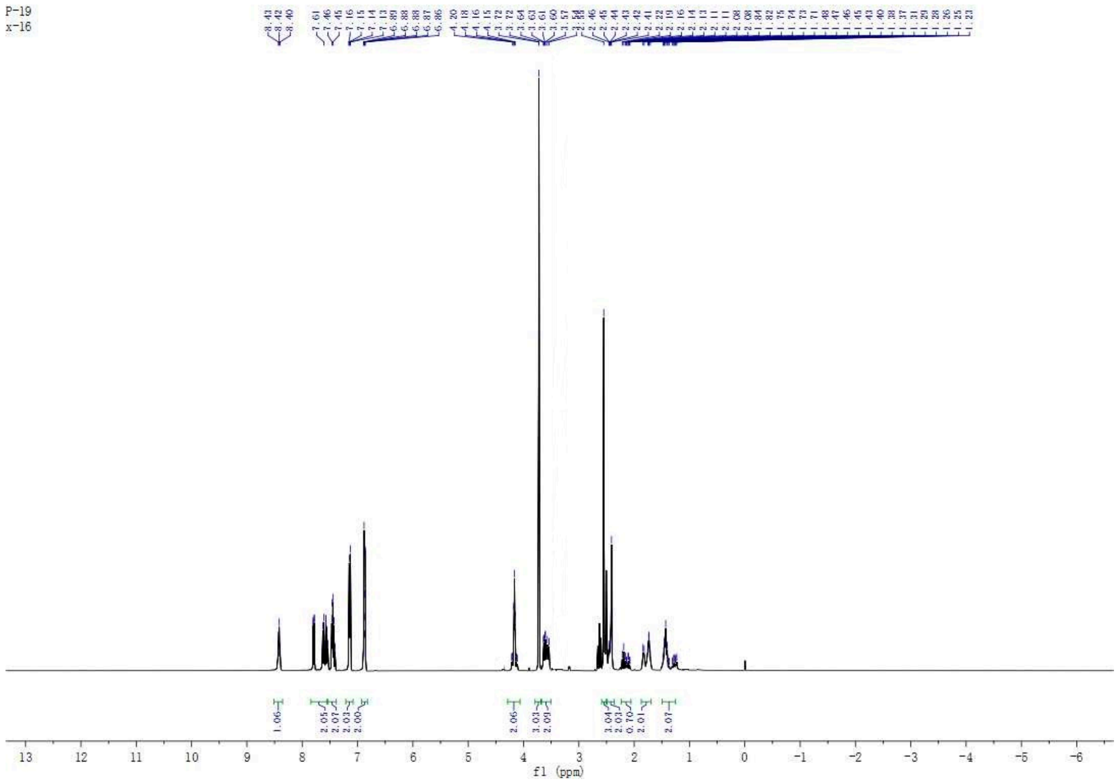

^{1}H NMR spectrum of compound H-19 (400 MHz, DMSO)

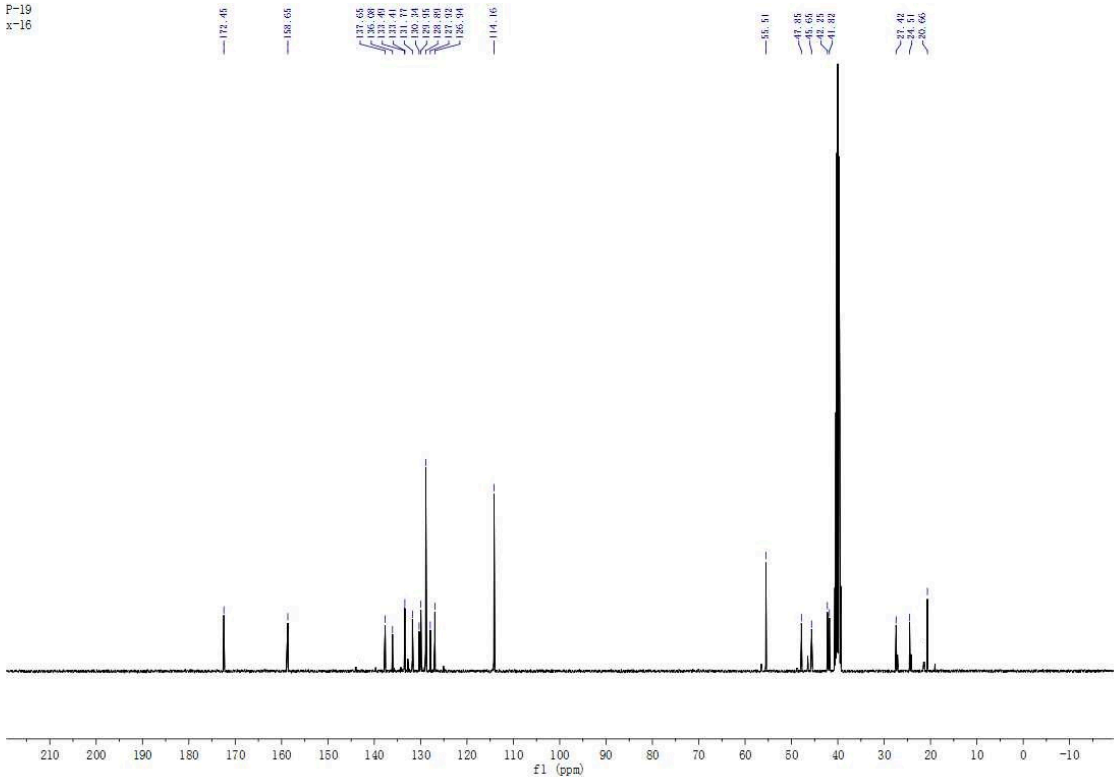

^{13}C NMR spectrum of compound H-19 (101 MHz, DMSO)

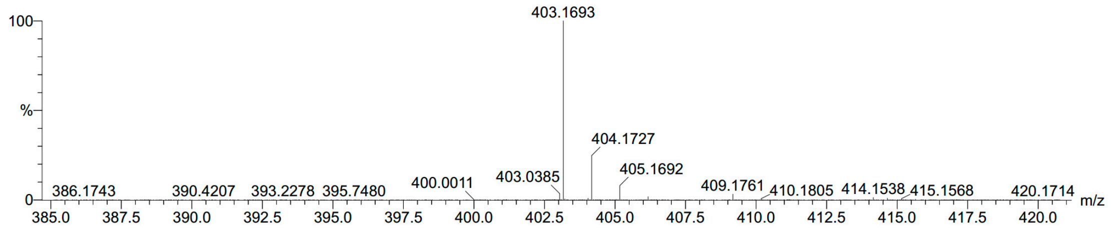

Minimum:				-1.5				
Maximum:		5.0	10.0	50.0				

Mass	Calc. Mass	mDa	PPM	DBE	i-FIT	Norm	Conf(%)	Formula
403.1693	403.1692	0.1	0.2	9.5	662.7	n/a	n/a	C21 H27 N2 04 S

High resolution mass spectrum of compound H-19

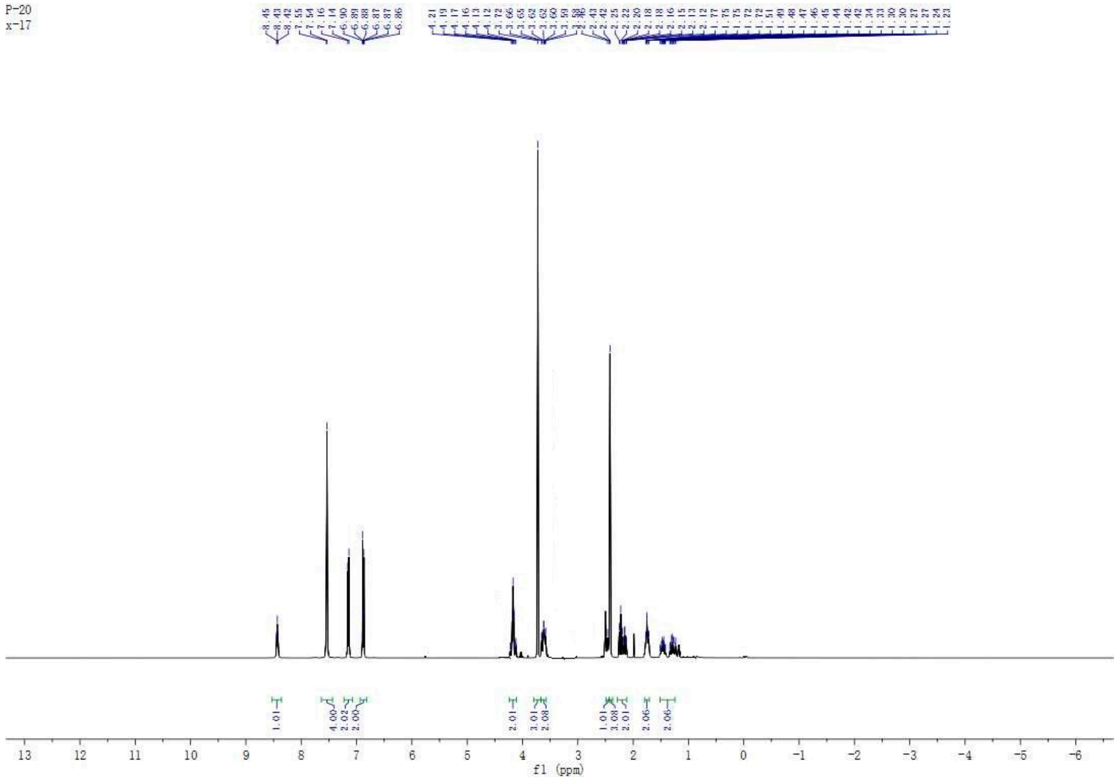

^{1}H NMR spectrum of compound H-20 (400 MHz, DMSO)

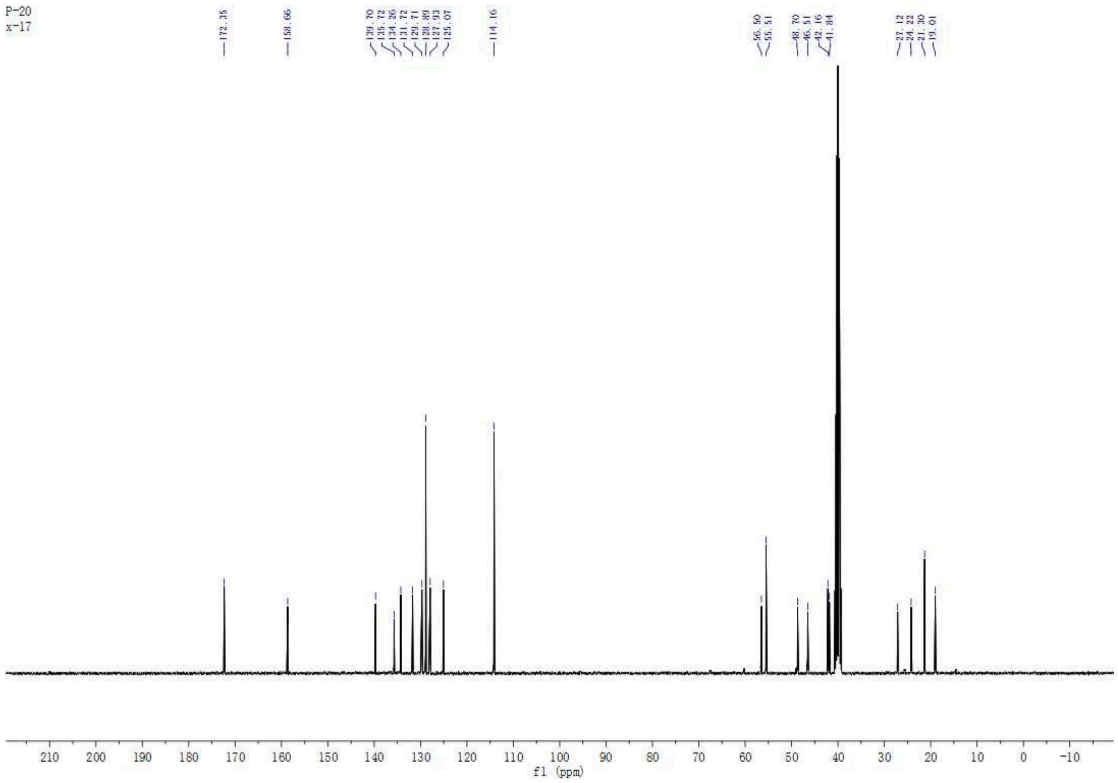

^{13}C NMR spectrum of compound H-20 (101 MHz, DMSO)

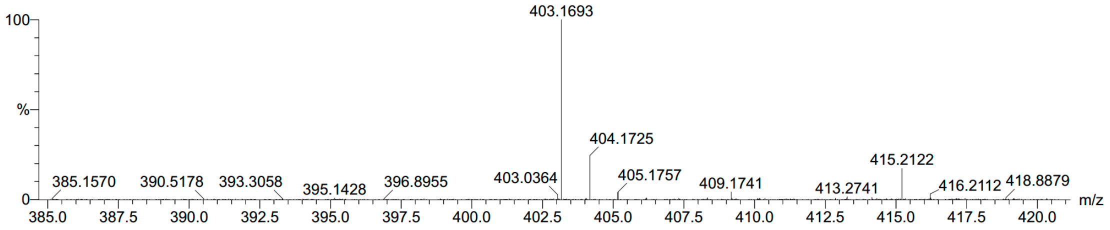

Minimum:				-1.5				
Maximum:		5.0	10.0	50.0				
Mass	Calc. Mass	mDa	PPM	DBE	i-FIT	Norm	Conf(%)	Formula
403.1693	403.1692	0.1	0.2	9.5	332.4	n/a	n/a	C21 H27 N2 O4 S

High resolution mass spectrum of compound H-20

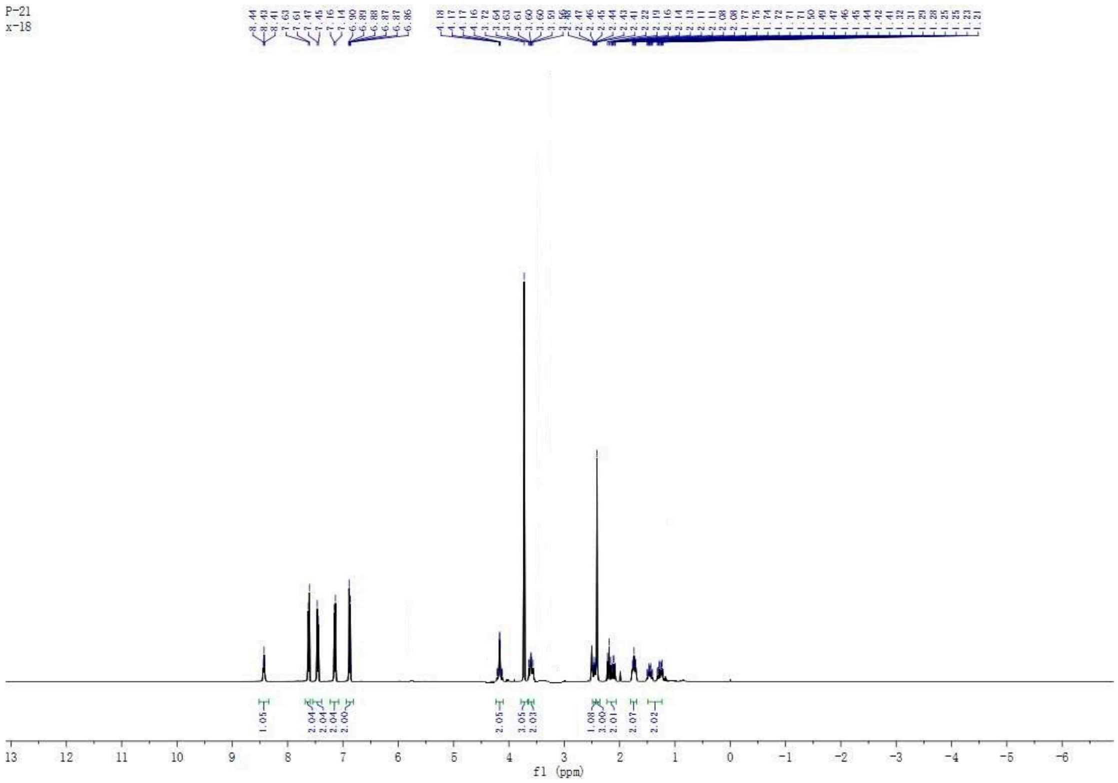

^{1}H NMR spectrum of compound H-21 (400 MHz, DMSO)

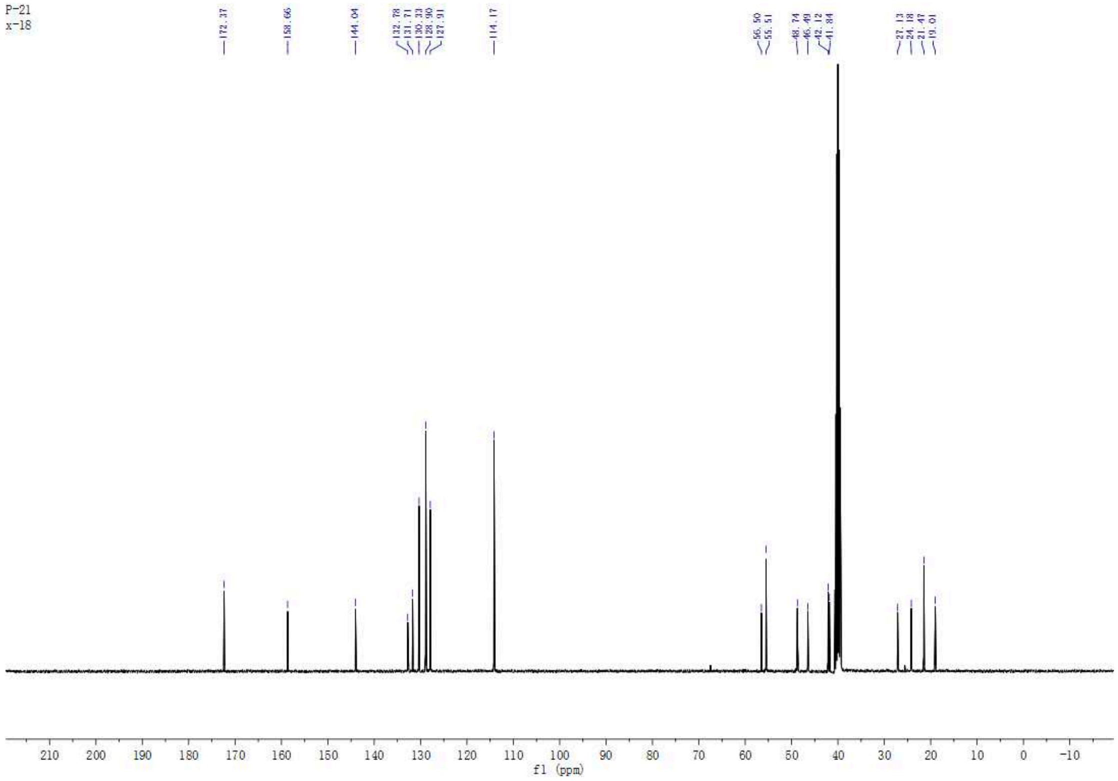

^{13}C NMR spectrum of compound H-21 (101 MHz, DMSO)

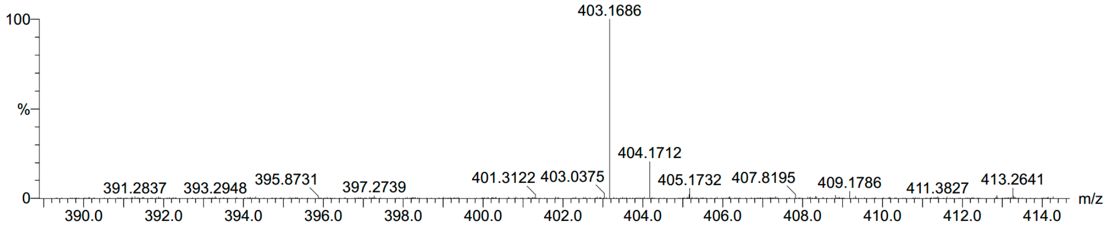

Minimum:				-1.5				
Maximum:		5.0	10.0	50.0				

Mass	Calc. Mass	mDa	PPM	DBE	i-FIT	Norm	Conf(%)	Formula
403.1686	403.1692	-0.6	-1.5	9.5	288.0	n/a	n/a	C21 H27 N2 O4 S

High resolution mass spectrum of compound H-21
